# Description and Analysis of the First Spectrum of Iodine

**DOI:** 10.6028/Jres.063A.001

**Published:** 1959-08-01

**Authors:** C. C. Kiess, C. H. Corliss

## Abstract

An extensive survey of the spectra of iodine has led to a list of more than 900 lines emitted by neutral atoms in the region from 23070 A in the infrared to 1195 A in the extreme ultraviolet. Wavelengths between 12304 A and 2061 A were derived from measurements of spectrograms obtained with gratings of high dispersion. Wavelengths of lines outside these limits are the computed values for lines observed on photometric tracings of the infrared, inaccessible to photography, and in the ultraviolet with a vacuum-grating spectrograph. For many of the lines Zeeman patterns were obtained in a magnetic field of about 37,000 oersteds. With these data many of the lines have been classified as combinations between odd levels from the electron configurations 5*s*^2^ *5p*^4^ *np* and 5*s*^2^ 5*p*^4^ *nf*, and even levels from the configurations 5*s*^2^ 5*p*^4^ *ns* and 5*s*^2^ 5*p*^4^ *nd.* Among these levels several sets have been recognized as forming Rydberg sequences that are in close agreement in placing the ground state 5*p*^5^ ${\;}^{2}P_{1½}^{o}$ of I i at 84,340 cm^−1^ below the ground state 5*p*^4 3^P_2_ of I ii. This gives 10.45 electron-volts for the ionization potential of the neutral iodine atom. A strong infrared line at 13148.8 A is explained as a magnetic dipole transition between the levels of the ground term 5*p*^5 2^P°.

## 1. Introduction

The spectra absorbed and emitted by iodine in its atomic and molecular states have been the object of many investigations. In volumes 5 and 8 of his Handbuch der Spectroscopie, Kayser lists 352 papers, which appeared up to 1933, dealing with various aspects of the spectral behavior of this heavy member of the halogen family. Since that date additional papers have appeared, of which some are cited below. But in spite of this abundant material, representing a vast amount of work, knowledge of the first spectrum of iodine, I i, emitted by neutral atoms, has remained scanty and fragmentary, largely owing to the fact that important parts of the spectrum lie in the not easily accessible ultraviolet and infrared regions. It is the purpose of this paper to present a new description of the first spectrum of iodine and an analysis of its term structure.

## 2. Experimental Procedure

The investigations of the first spectrum of iodine at the Bureau were made at two different times under different experimental conditions. The first series of observations was made more than 30 years ago when chlorine and bromine [1]^1^ also were being investigated. The light source was a Geissler tube of Pyrex glass into which a small amount of dry iodine vapor could be admitted from time-to-time to replace that which was adsorbed on the walls of the tube or absorbed by its aluminum electrodes. The lamp was similar to that used in the experiments on chlorine and bromine, and was excited to luminescence in the same way, with an uncondensed discharge from the high-voltage side of a 40-kv transformer. The spectrograms were recorded on plates sensitized to the green, orange, red, and infrared regions of the spectrum by bathing ordinary photoplates in solutions of the photosensitizing dyes available at that time; namely, pinaverdol, pinacyanol, dicyanin, and the newly discovered kryptocyanin. The spectrographs carried concave gratings of 21-ft radius of curvature, ruled with 7,500 and 20,000 lines per in. and set up in Wadsworth mountings. Each exposure to the light source was made with one-half the length of the spectrograph slit covered with a colored-glass filter so that both the first-order spectrum and the overlapping second order could be obtained at the same time. Each plate was exposed also to light from the iron arc, in both the first and second orders, to obtain the necessary standard wavelengths for use in deriving the iodine wavelengths. Because the capillary of the discharge became discolored after a run of a few hours, it was necessary to make exposures of nearly 24-hr duration in order to photograph the lines of longest wavelength recorded on the plates.

Measurement of these spectrograms yielded a list of approximately 400 wavelengths, with estimated intensities, extending from 9732 A in the infrared to 3820 A in the ultraviolet. This list was seemingly the most extensive description of the first spectrum of iodine then available and was being prepared for publication when the paper, “The Arc Spectrum of Iodine,” by Evans [2] appeared. A comparison of his list and ours showed that they were essentially identical. This fact and also the fact that his paper contains the first real results for a classification of the iodine spectrum outside of the Schumann region induced us to defer publication of our results until a substantial addition could be made to the description and analysis of the spectrum.

The second series of observations was made at various times during the period from 1953 to 1957. Improved apparatus and new experimental procedures have made it possible to advance the description of the iodine spectrum beyond the limits reached in the earlier work, and also to obtain Zeeman-effect observations that have led to a revision and extension of its term structure. The new light source was an electrodeless discharge tube of the type described by Corliss, Westfall, and Bozman [3]. It was excited to luminescence in a field of 2,450 Me from a microwave oscillator. The plates used to record the spectra were EK types 103a–O–UV, 103 a–O, I–F, I–N, I–Q, and I–Z, according to the region investigated, and, where required, were hypersensitized in an ammonia bath by the method recommended by Burka [4]. Four concave-grating spectrographs and a Hilger E 1 quartz-prism spectrograph were used to obtain the spectrograms. The spectrographs carrying the gratings with 7,500 and 15,000 lines per in. were used for the infrared and red regions where many new strong lines were found. For the shorter regions the grating with 30,000 lines per in. was used as well as the one with 15,000. For the extreme ultraviolet both the Hilger E 1 instrument and a 2-m glass grating ruled with 30,000 lines per in. and mounted in a vacuum chamber were used. All the spectrograms bore exposures to the iron arc or other sources of standard lines to be used in the determination of the iodine wavelengths.

For the Zeeman-effect observations the Weiss water-cooled magnet of the Bureau was used. With a current of 160 amp through the coils and a gap of 5 mm between the pole pieces, a field of approximately 37,000 oersteds was produced. The source between the pole pieces was also an electrodeless lamp of the type mentioned above, but of diameter 4 mm. A Wollaston prism of quartz placed between the light source and the projection lens of the spectrograph separated the two polarizations on the slit, with space between them for a no-field exposure. On plates appropriate for the regions under investigation resolved magnetic patterns were recorded for nearly all the strong lines of I i from 2062 A in the ultraviolet out to 11246 A in the infrared. Zeeman patterns were recorded also for some I ii lines of long wavelength.

## 3. Results

The observational data and the deductions from atomic theory that are essential for the description of the spectrum of iodine and its term structure are embodied in the tables appended to this paper. In tables 1, 2, and 3 are listed, in the first three columns, the wavelengths of the lines of I i, their estimated intensities and characteristics, and their wave numbers in vacuum. The letters after the intensity numbers have the following significance: *c*=partially resolved hyperfine structure (hfs); *d*=double; *w*=widened line owing to unresolved hfs; *h*=hazy, diffuse; *Z*=Zeeman pattern given in table 4. The letters *A* and *B* indicate the type of shading displayed by unresolved patterns; thus: *A*=⩘|\; *B*=|\/|. The term combinations in the fourth column of the tables are based on *g*- and *J*-values derived from the Zeeman-effect patterns of table 4.

Although the intensities are estimates made according to the usual practice of spectroscopists, an attempt has been made to bring them into closer relationship with photometric standards than is possible in a compressed linear scale in which the numbers are roughly proportional to the logarithms of the actual intensities. In table 2 the intensities are the measured heights of the peaks of the lines above the noise level of the recorder tracings.

In tables 1 and 3, however, an attempt has been made to bring the estimated intensities into harmony with a photometric scale that reflects the enormous range in the strength of the lines. On this scale the faintest lines are arbitrarily assigned an intensity 1, and the strongest lines, that occur in the multiplet 6*s*^4^P—6*p*^4^P° are designated as 10^5^ times as strong. This ratio is based on an accurate determination of the relative strengths of Rowland ghosts to their parent lines. Thus, for the strongest lines estimates were made of the intensities of their ghosts, and then multiplied by the corresponding factors to establish the intensities of the parent lines. Although the scale was thus established to represent true relative strengths of lines in short ranges of the spectrum, no attempt was made to standardize them over longer ranges of wavelengths. In particular the intensity scale used in the vacuum region is several orders of magnitude less than that of the visible and infrared. A few of the longest wavelengths listed in table 1 were measured also on the infrared recordings described above. The intensities derived for them from these observations are given in parentheses following the estimated photographic intensities, thereby giving a comparison of the relative sensitivity of the two modes of observation in a region near the limit of photographic detection.

The terms to be expected theoretically on the assumption that *LS*-coupling governs the orbital and spin angular momenta of the atom are given in table 5. Those actually found in this investigation are given in tables 6 and 7. These terms make it possible to calculate accurate wavelengths for lines in the infrared and extreme ultraviolet regions that lie beyond the reach of photographic recording with high-dispersion spectrographs. Such lines as have been observed by other investigators are given in tables 2 and 3 with wavelengths calculated from the terms of tables 6 and 7.

Lines listed in table 2 were observed by C. J. Humphreys [5] at the Naval Ordnance Laboratory, Corona, and by E. K. Plyler [6] at the Bureau. In this work they used electrodeless-discharge lamps, similar to those mentioned above in conjunction with their recording infrared spectrometers. These observations, which were made expressly for this investigation, verify all but two of the new infrared lines measured by Eshbach and Fisher [7] and add several lines not previously observed. All the ultraviolet lines in table 3 were measured on spectrograms obtained with the 2-m vacuum-grating spectrograph of the Bureau. These data not only confirm the descriptions of II given by Turner [8], La Croute [9], McLeod [10], and Hellerman [11], but increase by a factor of more than 4 the number of lines reported by these earlier observers. The light source was an electrodeless discharge of the type described above but modified to incorporate a LiF window sealed to the tube with an O-ring. The wavelengths listed in the first column are not the values derived directly from the measurements, but are values calculated from the energy levels given in tables 6 and 7. They were derived, therefore, indirectly from international secondary standard wavelengths, and are believed to be correct to less than 0.005 A. They are recommended for use as standards in the vacuum region.

A problem of prime importance for the analysis of the first spectrum of iodine is the evaluation of the separation of the levels of the ground state 5*p*^5 2^P°. The lines at 2061.6 and 1830.4 A, which are due to the transitions $5p^{5}{\;}^{2}P_{0½}^{o}—6s{\;}^{2}P_{1½}$ and $5p^{5}{\;}^{2}P_{1½}^{o}—6s{\;}^{4}P_{2½}$, may be used for this purpose. These lines have been measured several times by different observers, but the wavelengths reported for them are only approximately correct and are afflicted with the errors that are inherent in the reference lines against which they were measured. In the present work the mean wavelength of the longer line has been determined as 2061.633 A, from seven observations, made with high dispersion in the higher orders of the gratings, relative to international secondary standards in the iron arc spectrum.

The wavelength of the shorter line was determined as 1830.380 A from measurements relative to internal standards selected from the iodine spectrum itself. These lines are at 1876, 1844, 1799, 1702, and 1593 A, and appear with 1830 A on spectrograms made with the vacuum-grating spectrograph. We have determined accurate wavelengths for them, from measurements of lines of longer wavelength on high-dispersion spectrograms, by making use of the combination principle. Thus, we find the following level-separations, which are mean values of the wave-number differences between numerous pairs of well-measured lines:

$$\begin{array}{l} 6s{\;}^{4}P_{0½}—6s\;{\;}^{2}P_{1½}=4803.39\;{\text{cm}}^{-1}\\ 6s{\;}^{4}P_{1½}—6s\;{\;}^{2}P_{1½}=5726.93\;{\text{cm}}^{-1}\\ 6s{\;}^{2}P_{0½}—6s\;{\;}^{2}P_{1½}=7093.88\;{\text{cm}}^{-1}\\ 6s^{\prime }{\;}^{2}D_{1½}—6s\;{\;}^{2}P_{1½}=10262.33\;{\text{cm}}^{-1}\\ \;nd\;5.1_{1½}—6s\;{\;}^{2}P_{1½}=14262.05\;{\text{cm}}^{-1} \end{array}$$

By adding each of these numbers to 48489.73 cm^−1^, the wavenumber of 2061 A, we obtain the wavenumbers, and thence the exact wavelengths, of the selected standards. These are given in table 3.

With the accurate values thus established for the two transitions given above we have:

$$\begin{array}{l} \;1830.380\;\text{A is}\;5p^{5}{\;}^{2}P_{0½}^{o}—6s{\;}^{4}P_{2½}=54633.46\;{\text{cm}}^{-1}\\ \;6s{\;}^{4}P_{2½}—6s{\;}^{2}P_{1½}=14659.42\;{\text{cm}}^{-1}\\ \;1782.758\;\text{A is}\;5p^{5}{\;}^{2}P_{1½}^{o}—6s{\;}^{2}P_{1½}=\bar{56092.88}\;{\text{cm}}^{-1}\\ 2061.6338\;\text{A is}\;5p^{5}{\;}^{2}P_{0½}^{o}—6s{\;}^{2}P_{1½}=48489.73\;{\text{cm}}^{-1}\\ \;5p^{5}{\;}^{2}P_{1½}^{o}—5p^{5}{\;}^{2}P_{0½}^{o}=\bar{7603.15}\;{\text{cm}}^{-1}. \end{array}$$

## 4. Term Structure of I i

The spectrum to be expected theoretically for neutral iodine atoms, if *LS*-coupling governs their behavior under excitation, is that based on the terms given in table 5. In the unexcited state of the atom the electron configuration is 5*s*^2^ 5*p*^5^, which yields an inverted ^2^P term of odd parity. The higher states of even and odd parity arise when excitation of the atom leads to the electron configurations listed in the first column of table 5. Ionization of the atom leaves it in one of the states represented by the ^3^P, ^1^D, and ^1^S terms of the basic electron configuration 5*s*^2^ 5*p*^4^ of the ion I^+^. These three terms give rise, therefore, to the three families of terms that are likely to produce the strongest lines of the spectrum I i. A fourth family also is expected based on the addition of *s, p, d*, etc., electrons to the 5*p*^6^ configuration. The terms from these configurations will be doublets, and the lines arising from their combinations probably will be among the weaker lines of the spectrum.

The first real regularity in the spectrum of iodine was announced by Turner [12] who found the wavenumber interval of approximately 7,600 cm^−1^ recurring among several pairs of the lines observed by him in the Schumann region. Subsequently he correctly suggested that this difference represents the separation of the levels in the ground term ^2^P of the 5*s*^2^ 5*p*^5^ electron configuration. From this starting point all further advances in the interpretation of the first spectrum of iodine have been made. Evans [2], Deb [13], and Murakawa [14], in their analyses, have recognized these lines as resulting from the 6*s* → 5*p* transition, but they are not in agreement on their designations of the individual levels of the 5*p*^4^ 6*s* configuration. Inasmuch as the interpretations of I i offered by these investigators all rest on the 6*s*-levels it is, therefore, not surprising that there is disagreement among them.

The classifications of the lines of I i given in this paper likewise are based on the 6*s*-levels. With *g*- and *J*-values derived from well-resolved Zeeman patterns it is now possible to designate these levels with certainty and also some of the higher *np*-levels with which they combine. Increasing excitation of the iodine atoms brings into play still higher levels from the *np, nd, ns*, and *nf* electrons, but it is difficult to designate them with certainty because the *g*-values indicate a breakdown of the orbital and spin momenta to a coupling scheme between pure *LS*-coupling and *jj*-coupling, probably *jl*-coupling described by Racali [15].

Most of the levels of I i that are given in tables 6 and 7 result from the addition of *ns, np, nd*, and *nf* electrons to the lowest energy states ^3^P, ^1^D, and ^1^S of I ii. These were reported first by LaCroute [9] whose analysis, giving the relative positions of these terms, shows that the levels of ^3^P are separated by large differences in wave number. This fact has been an important guide in the analysis of I i, for nearly all the terms derived from ^3^P show similar characteristics. The eight levels of the 5*s*^2^ 5*p*^4^ 6*s* electron configuration all closely conform to the pattern of spacing exhibited by their parentage. This is confirmed by the *g*-values of these levels, which are nearly equal to the *g’s* for *LS*-coupling, their sum being $\sum_{g_{obs}}=11.985$ as compared with $\sum_{g_{LS}}=12.000$.

Of the levels coming from configurations with *np*, *nd*, and *nf* electrons, only those derived from ^3^P levels can be designated with certainty. Here again the classifications rest on level intervals and *g*-values; but the *g*-values now show marked deviations from *LS-g’*s, owing to configuration interaction, and there is a tendency for levels to form pairs. In the case of the 13 levels derived by adding a 6*p*-electron to the parent term ^3^P, *g*-sharing is such as to remove all resemblance to *LS g*-values, yet their sums are nearly equal, namely $\sum_{g_{obs}}=17.847$ and $\sum_{g_{LS}}=18.000$. Few if any levels from the parent terms, ^1^D and ^1^S, are definitely established.

## 5. Series and Ionization Potential

Before the first attempts were made to unravel the spectrum of iodine, several investigators reported the results of their measurements of critical and ionizing potentials in iodine vapor. Thus, in the period from 1920 to 1924, values of the ionization potential from 10 to 10.5 ev were reported by Found [16], Mohler and Foote [17], Duffendack [18], and Mackay [19]. From these results it was evident that the separation ${\;}^{2}P_{1½}^{o}-{\;}^{3}P_{2}$ between the ground states of I i and I ii is approximately 85,000 cm^−1^. Although the series announced by Deb [9] and by Price [20] give limits in close agreement with this value; yet, in the light of the present analysis, their series must be considered fictitious and the ionization potentials derived from them fortuitous.

The first physically real series of I i was given by Evans [2] in his analysis of the spectrum. Two of his levels in combination with the 6*s*-term accounted for two pairs of strong lines separated by the same difference in wave number. The limit and ionization potential derived from them are within the range of the experimental values cited above. This series has been confirmed and extended in the present work. Other series of three and more members have been found also, as set forth in table 8. Four of these series, with four and five members, representing the migration of the *s, p*, and *d* electrons, have been used to calculate the separation of the ground states of I i and I ii and the corresponding ionization potential. A Ritz formula *R*/(*m*+*α*+*β*/*m*^2^)^2^ has been evaluated for the variable terms of these series, and with the values given in the table for *α* and *β*, it is found to represent the series very closely. In fact the first solutions of this formula were found to fit series of three members closely enough to predict higher series members that were subsequently found. The individual determinations of the interval ${\;}^{2}P_{1½}^{o}-{\;}^{3}P_{2}$ between the ground states of I i and I ii are in close agreement among themselves, and when an unweighted average of them is taken, yield a value of 84,340 cm^−1^. From this an ionization potential of 10.45 ev is derived for the work required to remove a *p*-electron from the configuration 5*s*^2^ 5*p*^5^.

## 6. The Continuous Spectra

An outstanding feature of the spectra of iodine emitted by the electrodeless lamps used in this investigation is a succession of continua extending from 4800 A in the blue to 2900 A in the ultraviolet. These bands have been encountered by nearly all observers who have studied the spectra emitted by iodine molecules and atoms subjected to various modes of excitation. Perhaps the best description of them is that published by Curtis and Evans [21]. Our observations are in agreement with their view that these continua belong to two systems, the one consisting of a single band or of several broad overlapping bands between 4800 and 4035 A, the other consisting of a very broad strong band followed by several groups of much fainter and narrower bands. In each of these groups the individual bands are about 25 A in width, nearly equally spaced, and increase in intensity to a maximum near the center of the group and then decline. The mean separation of the bands is 210 cm^−1^, which is exactly the separation of the vibrational levels of the ground state of the I_2_ molecule as found by Kimura and Miyanashi [22] in their study of the ultraviolet absorption bands of I_2_. This fact casts doubt on the results of various investigators who have sought to ascribe the bands to atomic recombination processes. A description of these emission features as recorded on our spectrograms is given in table 9. Their presence is unwelcome in investigations of I i because they mask completely the fainter and widened lines and attenuate differences in intensity of measurable lines that appear on the continuous background.

## 7. Discussion

The analysis of the first spectrum of iodine, presented in the preceding pages, shows clearly that in its excited states the atom has departed from *LS*-coupling and has reached a stage in intermediate coupling. Since the *g*-value of $5p^{5}{\;}^{2}P_{0½}^{o}$, of the ground state, is very closely that given by *LS*-coupling, we may assume that the *g*-value of the lowest member of the ground term, $5p^{5}{\;}^{2}P_{1½}^{o}$, is also that for *LS*-coupling. In the first excited states, however, arising from the electron configuration 5*s*^2^ 5*p*^4^ 6*s*, the departure from *LS*-coupling is marked. Although the *g*-values deviate only slightly from the *LS*-values, the levels of the ^4^P and ^2^P terms show the separations that are characteristic of their parent term, ^3^P, the ground state of I ii. This is in accord with the scheme for the *p*^4^ configuration in intermediate coupling as illustrated by Condon and Shortley [23].

In the configurations containing *np*-electrons, in which *n* ≧ 6, almost all resemblance to *LS*-coupling has disappeared from the term structure. The *g*-values for all the levels deviate strongly from the *LS*-values, except those for ${\;}^{4}D_{3½}^{o}$ and ${\;}^{4}D_{2½}^{o}$ for which the deviation is slight. The levels fall into a pair structure, of the kind prescribed by Racah [15] for *jl*-coupling, in which the level separations bear no resemblance whatsoever to those of *LS*-coupling. The *g*-values calculated for the *np*-levels with Racah’s formula fit the observed *g*’s more closely than do the *g*’s of *LS*-coupling. A similar situation holds for levels from configurations with *nd*-electrons, and also with *nf*-electrons. The *nf*-levels fall very close to each other so that lines originating in them are separated by intervals less than those imposed by a magnetic field, say, of 35,000 oersteds. All of these lines for which magnetic patterns appear on our spectrograms of the Zeeman effect show the unsymmetrical structures, due to Paschen-Back interaction, similar to those described by Kiess and Shortley [24] for lines of oxygen and nitrogen.

These matters raise the question as to the appropriateness of the designations used for the energy levels of a heavy atom such as iodine. It is obvious that in a complex spectrum manifesting in its various states a transition from one coupling scheme to another, no single scheme of notation will be adequate or satisfactory. The only notation scheme that has achieved a status of widespread usage and permanence is the one devised for spectra built on *LS*-coupling. It is here emphasized that the use of it, in this paper, for levels that do not result from this coupling scheme, is for convenience in designating them, and not for attaching to them the quantum significance usually conveyed by the *LS*-symbols.

An inspection of table 1 will show that some lines have resisted all attempts to classify them. There is no doubt, however, that they belong to I i; but the necessary links to connect them to known or, as yet, unknown levels have not been found. One of the lines left unclassified after the bulk of the analysis had been completed is the relatively strong infrared line at 13149.19 A as measured by Eshback and Fisher [7]. This line, measured by us also on the recordings of the infrared spectrum of iodine by Plyler and by Humphreys, has a wave number practically identical with the separation of the levels in the ^2^P° ground term of the iodine atom. Accordingly it is designated in table 2 as the forbidden transition ${\;}^{2}P_{0½}^{o}\to {\;}^{2}P_{1½}^{o}$ and a wavelength corresponding to the wave number 7603.15 cm^−1^ is calculated for it. A similar transition has been reported by Edlén [25] for the isoelectronic spectrum Xe ii. We have tried to photograph this line on EK–IZ plates so as to get a more accurate value of its wavelength, but the experiments were unsuccessful. Our belief that our designation of it is correct is substantiated by the discovery of similar transitions between the metastable levels of I ii, as reported by Martin and Corliss in their forthcoming paper on I ii.

In conclusion we acknowledge our indebtedness to several of our colleagues for data used in this investigation. Both C. J. Humphreys and E. K. Plyler made observations of the infrared spectrum of iodine beyond the reach of photography. Their data are presented in table 3. W. F. Meggers and R. Zalubas measured the magnetic patterns of numerous iodine lines during their investigations of spectra emitted by various electrodeless metal-halide lamps. Finally, W. C. Martin, Jr., made new measurements of iodine spectra in the extreme ultraviolet that have surpassed in extent and accuracy earlier descriptions of these spectra. It is a pleasure for us to thank each for his contribution to this paper.

## Figures and Tables

**Table 1:** Wavelengths and term combinations of I i

Wave length	Intensity	Wave number	Designation

12304.77	10(435)	8124.71	6*p* ${\;}^{4}S_{1½}^{o}$*—nd* 6_1½_
12136.08	5*w* (180)	8237.65	*nd* 5. 1_1½_—*np* $2_{1½}^{o}$
12097.44	2	8263.96	*nd* 5_2½_—6*p*′ ${\;}^{2}P_{1½}^{o}$
12033.94	60	8307.57	6*p* ${\;}^{2}D_{3½}^{o}$*—nd* 10_3½_
12023.74	8	8314.61	6*s* ^2^P_0½_—6*p* ${\;}^{2}P_{0½}^{o}$
11996.92	75(510)	8333.20	6*p* ${\;}^{2}D_{2½}^{o}$*—nd* 10_3½_
11866.00	2	8425.14	*nd* 5.1_1½_—7*p* ${\;}^{2}P_{0½}^{o}$
11779.17	$\begin{array}{c} 30\\ 45 \end{array}\}(460)$	8487.24	6*p* ${\;}^{4}S_{1½}^{o}$*—nd* 7_1½_
11778.01		8488.08	
11761.74	2	8499.82	*nd* 5.1_1½_—8*p* ${\;}^{2}D_{2½}^{o}$
11650.40	1*w*	8581.05	*nd* 5_2½_—8*p* ${\;}^{4}P_{2½}^{o}$
11610.60	5	8610.47	6*s*′ ^2^D_1½_—7*p* ${\;}^{4}P_{2½}^{o}$
11588.23	40(365)	8627.09	6*s* ^2^P_0½_—6*p* ${\;}^{2}S_{1½}^{o}$
11558.56	100(660)	8649.23	6*p* ${\;}^{4}S_{1½}^{o}$*—nd* 8_1½_
11538.57	2	8664.22	*nd* 5_2½_—8*p* ${\;}^{4}S_{1½}^{o}$
11498.65	15	8694.30	6*s*′ ^2^D_1½_—7*p* ${\;}^{4}S_{1½}^{o}$
11486.80	8	8703.27	$\left\{ \begin{array}{l} 6p{\;}^{4}D_{1½}^{o}—7d{\;}^{4}P_{2½}\\ \;nd\;5_{2½}—{\;}^{2}D_{2½}^{o} \end{array}\right.$
11465.76	25*w*	8719.25	*nd* 3_2½_—4*f* $1_{2½}^{o}$
11458.07	6	8725.09	*nd* 3_2½_—4*f* $2_{3½}^{o}$
11457.08	8	8725.84	*nd* 3_2½_—4*f* $2.1_{3½}^{o}$
11451.13	50	8730.38	6*p* ${\;}^{4}P_{0½}^{o}$—*nd* 11_1½_
11447.72	100	8732.98	$\left\{ \begin{array}{l} 6p{\;}^{4}P_{2½}^{o}—nd\;8_{1½}\\ \;nd\;3_{2½}—3_{3½}^{o} \end{array}\right.$
11429.56	15	8746.86	
11428.40	50	8747.74	*nd* 4_1½_—4*f* $5_{2½}^{o}$
11420.33	75	8753.92	*nd* 2_1½_—4*f* $0.1_{0½}^{o}$
11415.66	3	8757.50	*nd* 2_1½_—4*f* $1_{2½}^{o}$
11410.07	75	8761.79	6*p* ${\;}^{4}P_{0½}^{o}$—*nd* 16_1½_
11401.46	2	8768.42	*nd* 3_2½_—4*f* $4_{1½}^{o}$
11397.98	10	8771.09	
11397.28	8	8771.63	*nd* 3_2½_—4*f* $5_{2½}^{o}$
11396.50	10*c*	8772.23	6*p* ${\;}^{2}D_{0½}^{o}$—*nd* 34_0½_
11375.25	75	8788.61	*nd* 4.1_1½_—4*f* $5.1_{1½}^{o}$
11373.78	1	8789.75	6*s* ^2^P_0½_*—*6*p* ${\;}^{4}D_{1½}^{o}$
11372.10	125	8791.05	*nd* 4.1_1½_—4*f* $6_{1½}^{o}$
11366.90	1	8795.07	*nd* 4_1½_—4*f* $6_{1½}^{o}$
11356.37	125	8803.23	
11353.67	75	8805.32	$\left\{ \begin{array}{l} 6p{\;}^{4}S_{1½}^{o}—nd9_{2½}\\ 6p{\;}^{4}D_{1½}^{o}—nd27_{1½} \end{array}\right.$
11351.81	18	8806.76	*nd* 2_1½_—4*f* $4_{1½}^{o}$
11347.85	100*d*	8809.84	*nd* 2_1½_—4*f* $5_{2½}^{o}$
11343.23	75	8813.42	6*s* ^2^P_1½_—6*p* ${\;}^{4}P_{2½}^{o}$
11313.27	100	8836.76	6*p* ${\;}^{4}P_{1½}^{o}$*—nd* 17_2½_
11298.66	4	8848.19	$\{\begin{array}{c} nd\;5.1_{1½}—8p{\;}^{4}P_{0½}^{o}\\ 6p\;{\;}^{4}D_{0½}^{o}—nd35_{0½} \end{array}$
11293.40	65*d*?	8852.32	*nd* 5_2½_—8*p* ${\;}^{4}D_{3½}^{o}$
11290.54	25*w*	8854.56	*nd* 2_1½_—4*f* $5.1_{1½}^{o}$
11287.49	75*d*	8856.95	*nd* 2_1½_*—*4*f* $6_{1½}^{o}$
11271.03	1	8869.88	*nd* 5.1_1½_—8*p* ${\;}^{4}P_{1½}^{o}$
11246.77	125	8889.02	$\{\begin{array}{c} 6p{\;}^{4}P_{2½}^{o}—nd\;9_{2½}\\ 6p{\;}^{2}D_{1½}^{o}—8d{\;}^{4}P_{2½} \end{array}$
11236.56	400*Z*	8897.09	6*s* ^2^P_1½_—6*p* ${\;}^{4}S_{1½}^{o}$
11187.21	5	8936.34	
11179.11	10	8942.82	6*p* ${\;}^{2}D_{2½}^{o}$*—nd* 11_1½_
11176.21	50	8945.14	6*s*′ ^2^D_2½_*—*7*p* ${\;}^{4}P_{2½}^{o}$
11172.79	8	8947.87	6*s*′ ^2^D_1½_—7*p* ${\;}^{4}P_{0½}^{o}$
11169.39	75	8950.60	
11147.15	10	8968.46	6*p* ${\;}^{2}S_{0½}^{o}$*—nd* 27_1½_
11140.20	6	8974.05	6*p* ${\;}^{2}P_{1½}^{o}$—*nd* 32.2_0½_
11138.10	2	8975.74	
11116.62	7	8993.09	
11093.70	4	9011.66	
11084.16	2	9019.42	6*p* ${\;}^{2}P_{1½}^{o}$*—ns* ^4^P_2½_
11072.33	60	9029.06	6*s*′ ^2^D_2½_—7*p* ${\;}^{4}S_{1½}^{o}$
11059.56	4	9039.48	$\{\begin{array}{c} 6p{\;}^{4}D_{0½}^{o}—nd36.1_{0½}\\ 6p{\;}^{2}P_{1½}^{o}—nd33_{0½} \end{array}$
11053.79	15	9044.20	6*p* ${\;}^{4}P_{1½}^{o}$*—nd* 18_2½_
11050.04	2	9047.27	
11020.60	250*Z*	9071.44	6*p* ${\;}^{4}P_{2½}^{o}$*—nd* 10_3½_
11017.14	100*Z*	9074.29	6*p* ${\;}^{4}P_{1½}^{o}$*—nd* 19_1½_
10991.19	4	9095.71	6*p* ${\;}^{4}D_{1½}^{o}$*—nd* 28.2_1½_
10979.70	6	9105.23	6*p* ${\;}^{2}P_{1½}^{o}$*—nd* 34_0½_
10970.39	4	9112.95	6*p* ${\;}^{4}D_{2½}^{o}$*—nd* 31_1½_
10914.32	15	9159.77	6*p* ${\;}^{4}D_{2½}^{o}$—8*d* ^4^D_3½_
10897.87	65	9173.60	6*s*′ ^2^D_2½_—7*p* ${\;}^{4}D_{3½}^{o}$
10894.66	70	9176.30	
10891.47	75*d*?	9178.99	$\{\begin{array}{c} 6p{\;}^{2}P_{0½}^{o}—7d{\;}^{4}P_{2½}\\ 6p{\;}^{2}D_{2½}^{o}—nd13_{2½} \end{array}$
10889.23	18	9180.88	6*p* ${\;}^{2}P_{1½}^{o}$—*nd* 35_0½_
10856.80	2	9208.31	6*p* ${\;}^{4}D_{2½}^{o}$—*nd* 32_1½_
10841.34	4	9221.43	6*p* ${\;}^{2}D_{1½}^{o}$—*nd* 32.2_0½_
10788.18	10	9266.87	$\{\;\begin{array}{c} 6s^{\prime }{\;}^{2}D_{1½}—7p{\;}^{4}P_{1½}^{o}\\ 6p{\;}^{2}D_{1½}^{o}—7p{\;}^{4}P_{2½} \end{array}$
10777.97	4	9275.65	6*p* ${\;}^{4}D_{1½}^{o}$—10s ^4^P_2½_
10777.79	20	9280.98	6*p* ${\;}^{2}P_{0½}^{o}$—*nd* 27_1½_
10722.07	2	9324.01	7*s* ^4^P_2½_*—*6*f* $6_{1½}^{o}$
10706.79	15	9337.31	
10696.02	100	9346.72	*nd* 5.1_1½_—8*p* ${\;}^{2}P_{0½}^{o}$
10685.82	100*Z*	9355.64	6*p* ${\;}^{4}P_{1½}^{o}$—*nd* 19.1_0½_
10603.64	4	9428.15	$\{\;\begin{array}{c} 6p{\;}^{2}D_{1½}^{o}—nd\;35_{0½}\\ 7s{\;}^{2}P_{1½}—7p{\;}^{4}D_{1½}^{o} \end{array}$
10588.59	6	9441.55	6*p* ${\;}^{2}P_{1½}^{o}$*—nd* 38_0½_
10578.22	20	9450.80	
10545.62	15	9480.02	*nd* 5.1_1½_—5*f* $1_{1½}^{o}$
10539.72	50	9485.33	*nd* 5.1_1½_—5*f* $2_{0½}^{o}$
10534.95	10	9489.62	*nd* 5.1_1½_—5*f* $3_{2½}^{o}$
10515.40	100	9507.27	6*p* ${\;}^{4}D_{3½}^{o}$*—nd* 14_2½_
10494.08	30 *w, + g*?	9526.58	*nd* 5.1_1½_—5*f* $5_{1½}^{o}$
10487.23	10	9532.80	6*p* ${\;}^{2}D_{2½}^{o}$*—nd* 14_2½_
10469.23	3	9549.19	
10466.54	5000*Z*	9551.65	6*s* ^2^P_1½_—6*p* ${\;}^{2}D_{2½}^{o}$
10459.55	1	9558.03	
10455.45	2	9561.77	
10445.35	5	9571.03	6*p* ${\;}^{2}P_{0½}^{o}$—*nd* 28.2_1½_
10438.81	3, + *g*?	9577.02	
10435.34	100d?*Z*	9580.10	
10428.39	6	9586.60	6*p* ${\;}^{2}P_{1½}^{o}$—*nd* 39_2½_
10416.61	75*Z*	9597.44	6*p* ${\;}^{2}S_{1½}^{o}$—*nd* 11_1½_
10412.80	10	9600.95	6*s*′ ^2^D_2½_—7*p* ${\;}^{4}P_{1½}^{o}$
10391.74	400*Z*	9620.40	$\{\;\begin{array}{c} 6s{\;}^{2}P_{0½}—6p{\;}^{2}D_{1½}^{o}\\ np\;1_{1½}^{o}—nd\;38_{0½} \end{array}$
10375.20	400*Z*	9635.74	
10354.93	8	9654.60	
10348.02	7	9661.04	6*p* ${\;}^{4}D_{0½}^{o}$—6*s*″ ${\;}^{2}S_{0½}^{o}$
10343.20	3	9665.54	6*p* ${\;}^{4}D_{1½}^{o}$—*nd* 31_1½_
10326.53	75	9681.15	6*p* ${\;}^{4}P_{2½}^{o}$—*nd* 11_1½_
10325.90	100*Z*	9681.74	6*s* ^4^P_1½_—6*p* ${\;}^{2}P_{0½}^{o}$
10322.56	100	9684.87	$\{\;\begin{array}{c} 6p{\;}^{2}D_{1½}^{o}—nd\;37_{2½}\\ nd\;5.1_{1½}—np\;3_{1½}^{o}\\ 6s{\;}^{2}P_{1½}^{o}—nd\;20_{0½} \end{array}$
10318.20	35	9688.96	$\{\;\begin{array}{c} 6s{\;}^{2}P_{0½}—np\;1_{1½}^{o}\\ 6p{\;}^{2}D_{1½}^{o}—\text{nd}\;38_{0½} \end{array}$
10313.72	4	9693.17	*nd* 5_2½_—5*f* $3_{2½}^{o}$
10310.20	50	9696.48	*nd* 5_2½_—5*f* $4_{3½}^{o}$
10286.07	8	9719.23	6*p* ${\;}^{4}D_{1½}^{o}$—8*d* ^4^P_2½_
10274.34	4*w*	9730.32	*nd* 5_2½_—5*f* $5_{1½}^{o}$
10266.04	5	9738.19	
10242.83	10	9760.26	6*p* ${\;}^{4}D_{2½}^{o}$—9*d* ^4^D_3½_
10241.29	20	9761.73	
10238.82	1000*Z*	9764.08	6*s* ^2^P_1½_—6*p* ${\;}^{4}P_{0½}^{o}$
10232.06	35	9770.53	*nd* 5.1_1½_—*np* $4_{2½}^{o}$
10211.60	5	9790.11	7*s* ^4^P_2½_*—np* $5_{3½}^{o}$
10201.82	7	9799.49	*nd* 4.1_1½_—6*p*′ ${\;}^{2}P_{1½}^{o}$
10172.91	300*Z*	9827.34	*nd* 3_2½_—6*p*′ ${\;}^{2}P_{1½}^{o}$
10166.00	8	9834.02	$\{\;\begin{array}{c} 6p{\;}^{2}D_{1½}^{o}—nd\;39_{2½}\\ 6p{\;}^{4}S_{1½}^{o}—nd\;13_{2½} \end{array}$
10158.64	400*Z*	9841.15	$\{\;\begin{array}{c} 6p{\;}^{4}P_{1½}^{o}—nd\;21_{2½}\\ 6p{\;}^{4}D_{3½}^{o}—5d\;{\;}^{4}D_{3½} \end{array}$
10147.70	1	9851.77	6*p* ${\;}^{2}S_{0½}^{o}$—*nd* 30_0½_
10141.83	100*Z*	9857.47	6*p* ${\;}^{4}P_{0½}^{o}$—*nd* 15_0½_
10133.56	40	9865.51	*nd* 2_1½_—6*p*′ ${\;}^{2}P_{1½}^{o}$
10132.38	3	9866.66	6*p* ${\;}^{2}D_{2½}^{o}$—5*d* ^4^D_3½_
10131.16	750*Z*	9867.85	6*s* ^2^P_0½_—6*p* ${\;}^{2}P_{1½}^{o}$
10126.07	7	9872.81	6*p* ${\;}^{2}P_{0½}^{o}$—*nd* 29_1½_
10109.70	5	9888.79	*nd* 5_2½_*—np* $3_{1½}^{o}$
10074.13	7	9923.71	*nd* 4_1½_*—np* $1.1_{2½}^{o}$
10066.72	7	9931.01	6*p* ${\;}^{2}S_{0½}^{o}$*—nd* 32.1_0½_
10050.11	2*w*	9947.42	*nd* 3_2½_*—np* $1.1_{2½}^{o}$
10034.64	2	9962.76	6*p* ${\;}^{4}D_{2½}^{o}$*—nd* 37_2½_
10030.35	2	9967.03	6*p* ${\;}^{4}P_{0½}^{o}$*—nd* 16_1½_
10023.10	22	9974.24	*nd* 5_2½_*—np* $4_{2½}^{o}$
10011.68	20	9985.61	*nd* 2_1½_*—np* $1.1_{2½}^{o}$
10003.05	500*Z*	9994.22	$\{\;\begin{array}{c} 6s{\;}^{4}P_{1½}—6p{\;}^{2}S_{0½}^{o}\\ 6p{\;}^{2}P_{1½}^{o}—6s^{\prime \prime }{\;}^{2}S_{0½} \end{array}$
9992.54	85	10004.72	*nd* 3_2½_*—np* $2_{1½}^{o}$
9992.24	4	10005.02	*nd* 1_1½_—4*f* $0.1_{0½}^{o}$
9963.30	400*Z*	10034.09	6*p* ${\;}^{4}D_{3½}^{o}$—5*d* ^4^F_4½_
9954.63	200*d*	10042.82	*nd* 2_1½_*—np* $2_{1½}^{o}$
9939.30	100	10058.31	*nd* 1_1½_—4*f* $4_{1½}^{o}$
9933.25	8	10064.44	
9892.35	50	10106.05	*nd* 1_1½_—*4f* $5.1_{1½}^{o}$
9889.95	40*c*	10108.46	*nd* 1_1½_—4*f* $6_{1½}^{o}$
9855.04	1	10144.31	*nd* 3_2½_—8*p* ${\;}^{4}P_{2½}^{o}$
9842.75	150*Z*	10156.98	6*s* ^4^P_1½_—6*p* ${\;}^{4}D_{1½}^{o}$
9835.52	5	10164.45	6*p* ${\;}^{2}P_{0½}^{o}$*—nd* 30_0½_
9832.28	4	10167.80	*nd* 4_1½_*—*7*p* ${\;}^{2}P_{0½}^{o}$
9827.53	1	10172.71	*np* $1_{1½}^{o}$—6*s*″ ^2^S_0½_
9818.10	2	10182.48	*nd* 2_1½_—8*p* ${\;}^{4}P_{2½}^{o}$
9817.59	1	10183.01	6*p* ${\;}^{4}D_{1½}^{o}$*—nd* 34_0½_
9813.53	200*Z*	10187.22	6*p* ${\;}^{4}S_{1½}^{o}$—*nd* 14_2½_
9808.26	1	10192.70	6*p* ${\;}^{2}P_{0½}^{o}$*—nd* 31.1_1½_
9800.89	100*Z*	10200.36	6*s* ^2^P_0½_—6*p* ${\;}^{4}D_{0½}^{o}$
9787.21	2	10214.62	6*p* ${\;}^{2}S_{0½}^{o}$*—nd* 32.2_0½_
9774.97	10*c*	10227.41	*nd* 3_2½_—8*p* ${\;}^{4}S_{1½}^{o}$
9773.50	6	10228.94	6*p* ${\;}^{4}D_{3½}^{o}$*—nd* 17_2½_
9772.17	7*c*	10230.34	*nd* 2_1½_*—*7*p* ${\;}^{2}P_{0½}^{o}$
9749.20	40*cZ*	10254.44	6*p* ${\;}^{2}D_{2½}^{o}$*—nd* 17_2½_
9747.23	3*c*	10256.51	
9733.56	300	10270.92	6*p* ${\;}^{4}P_{2½}^{o}$*—nd* 14_2½_
9731.73	5000*Z*	10272.85	6*s* ^4^P_2½_—6*p* ${\;}^{4}P_{2½}^{o}$
9725.47	30*Z*	10279.46	6*p* ${\;}^{4}P_{0½}^{o}$*—nd* 19_1½_
9710.58	8	10295.22	
9701.28	5	10305.09	*nd* 2_2½_—8*p* ${\;}^{2}D_{2½}^{o}$
9673.43	1	10334.76	6*p* ${\;}^{4}D_{3½}^{o}$—5*d* ^2^F_3½_
9663.06	1	10345.86	6*p* ${\;}^{2}S_{0½}^{o}$*—nd* 34_0½_
9653.06	3000*dZ*	10356.57	6*s* ^4^P_2½_—6*p* ${\;}^{4}S_{1½}^{o}$
9649.61	2000*Z*	10360.27	6*p* ${\;}^{2}D_{2½}^{o}$—5*d* ^2^F_3½_
9623.21	15	10388.69	6*p* ${\;}^{4}P_{1½}^{o}$—8*s* ^4^P_2½_
9598.22	2000*Z*	10415.74	*nd* 3_2½_—8*p* ${\;}^{4}D_{3½}^{o}$
9593.62	2	10420.74	6*p* ${\;}^{4}D_{1½}^{o}$*—nd* 36_2½_
9579.02	20*c*	10436.62	6*p* ${\;}^{4}D_{3½}^{o}$*—nd* 18_2½_
9573.22	1	10442.94	*nd* 5.1_1½_—9*p* ${\;}^{2}D_{2½}^{o}$
9566.81	1	10449.94	6*p* ${\;}^{4}D_{1½}^{o}$—*nd* 36.1_0½_
9555.72	10	10462.07	6*p* ${\;}^{2}D_{2½}^{o}$*—nd* 18_2½_
9528.48	18	10491.98	6*p* ${\;}^{2}D_{2½}^{o}$*—nd* 19_1½_
9526.90	30	10493.72	6*p* ${\;}^{4}P_{1½}^{o}$—8*s* ^2^P_1½_
9516.92	1*h*	10504.72	
9514.07	1	10507.86	
9496.69	1	10527.09	6*p* ${\;}^{2}P_{0½}^{o}$*—nd* 32.2_0½_
9466.34	50*cZ*	10560.85	6*p* ${\;}^{4}P_{0½}^{o}$*—nd* 19.1_0½_
9438.25	1*h*	10592.28	6*p* ${\;}^{2}P_{0½}^{o}$*—nd* 33_0½_
9427.15	3000*Z*	10604.75	6*p* ${\;}^{4}P_{2½}^{o}$—5*d* ^4^D_3½_
9426.71	4000*Z*	10605.25	6*s* ^4^P_0½_—6*p* ${\;}^{4}P_{0½}^{o}$
9423.42	1	10608.94	*nd* 4.1_1½_—8*p* ${\;}^{4}P_{1½}^{o}$
9398.73	200*c*	10636.82	*nd* 3_2½_—8*p* ${\;}^{4}P_{1½}^{o}$
9390.14	1	10646.55	*nd* 5_2½_—9*p* ${\;}^{2}D_{2½}^{o}$
9379.77	2	10658.32	6*p* ${\;}^{2}P_{0½}^{o}$—*nd* 34_0½_
9374.34	*3d*	10664.49	6*p* ${\;}^{4}D_{1½}^{o}$—*nd* 39_2½_
9365.16	25*c*	10674.95	*nd* 2_1½_—8*p* ${\;}^{4}P_{1½}^{o}$
9358.69	1	10682.33	6*p* ${\;}^{2}S_{0½}^{o}$—*nd* 38_0½_
9335.05	1000*Z*	10709.38	6*s* ^4^P_1½_—6p ${\;}^{4}D_{2½}^{o}$
9321.95	200*Z*	10724.43	6*p* ${\;}^{4}S_{1½}^{o}$—*nd* 15_0½_
9313.64	5	10734.02	6*p* ${\;}^{2}P_{0½}^{o}$—*nd* 35_0½_
9227.74	600*Z*	10833.92	6*p* ${\;}^{4}S_{1½}^{o}$*—nd* 16_1½_
9213.57	3	10850.58	*nd* 5.1_1½_—6*f* $1_{2½}^{o}$
9201.90	12	10864.33	*nd* 5.1_1½_—6*f* $4_{2½}^{o}$
9195.07	3*d*	10872.40	*nd* 5.1_1½_—6*f* $6_{1½}^{o}$
9180.20	70*Z*	10890.02	6*p* ${\;}^{4}P_{0½}^{o}$—*nd* 20_0½_
9164.38	20	10908.82	6*p* ${\;}^{4}S_{1½}^{o}$*—nd* 17_2½_
9156.91	500*Z*	10917.72	$\{\;\begin{array}{c} 6s{\;}^{4}P_{0½}—6p{\;}^{2}S_{0½}^{o}\\ 6p{\;}^{4}P_{2½}^{o}—nd16_{1½} \end{array}$
9150.63	3	10925.21	6*p* ${\;}^{2}P_{0½}^{o}$—*nd* 36.1_0½_
9128.03	600*Z*	10952.26	6*s*′ ^2^D_1½_—4*f* $1_{2½}^{o}$
9113.91	12000*Z*	10969.23	6*s* ^2^P_1½_—6*p* ${\;}^{4}P_{1½}^{o}$
9098.86	1000*Z*	10987.37	6*s* ^4^P_1½_—6*p* ${\;}^{2}D_{1½}^{o}$
9094.50	150	10992.64	6*p* ${\;}^{4}P_{2½}^{o}$*—nd* 17_2½_
9087.16	300*Z*	11001.52	6*s*′ ^2^D_1½_—4*f* $4_{1½}^{o}$
9084.70	6	11004.50	6*s*′ ^2^D_1½_—4*f* $5_{2½}^{o}$
9082.55	2	11007.10	6*p* ${\;}^{2}P_{0½}^{o}$—*nd* 38.2_1½_
9079.34	50*Z*	11010.99	6*s* ^4^P_2½_—6*p* ${\;}^{2}D_{2½}^{o}$
9058.33	15000*Z*	11036.53	6*s* ^4^P_2½_—6*p* ${\;}^{4}D_{3½}^{o}$
9047.91	20*c*	11049.24	6*s*′ ^2^D_1½_—4*f* $5.1_{1½}^{o}$
9046.90	3	11050.47	
9046.36	5	11051.13	
9043.85	1	11054.20	*nd* 5_2½_—6*f* $1_{2½}^{o}$
9042.30	15	11056.10	$\{\;\begin{array}{c} 6s{\;}^{4}P_{1½}—np\;1_{1½}^{o}\\ nd\;5_{2½}—6f\;2_{3½}^{o} \end{array}$
9034.43	2*d*?	11065.73	*nd* 5_2½_—6*f* $3_{1½}^{o}$
9032.64	3	11067.92	*nd* 5_2½_—6*f* $4_{2½}^{o}$
9022.40	5000*Z*	11080.48	6*s* ^4^P_0½_—6*p* ${\;}^{4}D_{1½}^{o}$
9018.05	10*c*	11085.83	*nd* 4.1_1½_—8*p* ${\;}^{2}P_{0½}^{o}$
9007.77	40	11098.48	6*p* ${\;}^{4}P_{2½}^{o}$—5*d* ^2^F_3½_
9004.39	6*c*	11102.65	
8993.13	400*Z*	11116.55	6*p* ${\;}^{4}S_{1½}^{o}$—*nd* 18_2½_
8969.04	300*Z*	11146.41	6*p* ${\;}^{4}S_{1½}^{o}$—*nd* 19_1½_
8964.69	400*Z*	11151.81	$\left\{ \begin{array}{c} nd\;5.1_{1½}—7p{\;}^{2}S_{0½}^{o}\\ nd\;2_{1½}—8p{\;}^{2}P_{0½}^{o} \end{array}\right.$
8925.97	225*cZ*	11200.20	6*p* ${\;}^{4}P_{2½}^{o}$—*nd* 18_2½_
8902.23	60*c*	11230.06	6*p* ${\;}^{4}P_{2½}^{o}$—*nd* 19_1½_
8899.72	3	11233.23	6*p* ${\;}^{4}D_{3½}^{o}$—*nd* 21_2½_
8898.50	1000*dZ*	11234.77	$\{\;\begin{array}{c} 6p{\;}^{2}S_{0½}^{o}—6s^{\prime \prime }{\;}^{2}S_{0½}^{o}\\ 6s{\;}^{4}P_{1½}—6p{\;}^{2}P_{1½}^{o} \end{array}$
8884.70	30*c*	11252.21	
8879.55	90*d*	11258.74	6*p* ${\;}^{2}D_{2½}^{o}$*—nd* 21_2½_
8878.76	75*c*	11259.74	*nd* 3_2½_—5*f* $4_{3½}^{o}$
8874.18	25	11265.56	*nd* 3_2½_—5*f* $4.1_{3½}^{o}$
8873.86	30*d*	11265.97	6*p* ${\;}^{4}D_{3½}^{o}$—*nd* 21.1_3½_
8870.66	6	11270.03	*nd* 41_1½_—5*f* $5_{1½}^{o}$
8868.00	75	11273.41	
8865.53	1	11276.54	6*s*′ ^2^D_2½_—4*f* $0_{1½}^{o}$
8864.95	1*c*	11277.28	*nd* 3_2½_—5*f* $4.2_{3½}^{o}$
8862.33	1	11280.61	
8858.79	3*c*	11285.12	*nd* 2_1½_—5*f* $1_{1½}^{o}$
8857.50	3000*Z*	11286.77	6*s*′ ^2^D_2½_—4*f* $1_{2½}^{o}$
8854.68	1*c*	11290.36	*nd* 2_1½_—5*f* $2_{0½}^{o}$
8853.80	2000*Z*	11291.49	6*p* ${\;}^{2}D_{2½}^{o}$—*nd* 21.1_3½_
8853.24	1000*Z*	11292.20	6*s*′ ^2^D_2½_—4*f* $2_{3½}^{o}$
8852.65	50*d*	11292.95	6*s*′ ^2^D_2½_—4*f* $2.1_{3½}^{o}$
8851.30	100*c*	11294.68	*nd* 2_1½_—5*f* $3_{2½}^{o}$
8848.33	80	11298.47	
8847.14	250*Z*	11299.99	6*s*′ ^2^D_2½_—4*f* $3_{3½}^{{}^{\circ}}$
8822.08	50*c*	11332.09	*nd* 2_2½_—5*f* $5_{1½}^{o}$
8818.89	150*c*	11336.18	6*s*′ ^2^D_2½_*—*4*f* $4_{1½}^{o}$
8816.65	275*cZ*	11339.07	6*s*′ ^2^D_2½_—4*f* $5_{2½}^{o}$
8812.40	100*d*	11345.82	
8809.86	10	11347.80	
8805.41	15*d*	11353.54	6*p* ${\;}^{4}P_{1½}^{{}^{\circ}}$—7*s* ^4^P_0½_
8780.10	100*c*	11386.27	6*s*′ ^2^D_2½_—4*f* $6_{1½}^{{}^{\circ}}$
8748.22	250*dZ*	11427.76	$\{\;\begin{array}{c} 6p{\;}^{4}S_{1½}^{o}—nd19.1_{0½}\\ nd\;4_{1½}—np3_{1½}^{o} \end{array}$
8729.70	200*c*	11452.01	*nd* 3_2½_*—np* $3_{1½}^{{}^{\circ}}$
8700.80	500*cZ*	11490.04	*nd* 2_1½_*—np* $3_{1½}^{{}^{\circ}}$
8680.36	10*d*	11517.10	*nd* 1_1½_—8*p* ${\;}^{4}S_{1½}^{{}^{\circ}}$
8664.95	1500*cZ*	11537.60	*nd* 3_2½_—*np* $4_{2½}^{{}^{\circ}}$
8642.60	200*d*	11567.42	6*s* ^4^P_1½_—6*p* ${\;}^{4}D_{0½}^{{}^{\circ}}$
8636.40	175*dZ*	11575.72	*nd* 2_1½_*—np* $4_{2½}^{{}^{\circ}}$
8560.30	30*d*	11678.63	*nd* 5.1_1½_—7*p* ${\;}^{4}D_{2½}^{{}^{\circ}}$
8551.60	1*cw*	11690.51	
8545.52	300*dZ*	11698.82	6*p* ${\;}^{4}P_{0½}^{{}^{\circ}}$—8*s* ^2^P_1½_
8503.32	2*cw*	11756.89	6*p* ${\;}^{4}S_{1½}^{{}^{\circ}}$*—nd* 20_0½_
8490.67	1*cw*	11774.40	
8486.11	1000*Z*	11780.73	6*p* ${\;}^{4}D_{3½}^{{}^{\circ}}$—8*s* ^4^P_2½_
8467.80	5	11806.21	6*p* ${\;}^{2}D_{2½}^{{}^{\circ}}$—8*s* ^4^P_2½_
8451.46	60*d*	11829.03	6*p* ${\;}^{4}P_{1½}^{{}^{\circ}}$—7*s* ^4^P_1½_
8443.19	8*c*	11840.61	
8427.41	20*c*	11862.80	6*s* ^2^P_0½_—7*p* ${\;}^{4}S_{1½}^{{}^{\circ}}$
8418.95	4	11874.71	*nd* 5_2½_—7*f* $1_{2½}^{{}^{\circ}}$
8416.54	50	11878.11	
8414.32	40	11881.24	6*p* ${\;}^{4}P_{1½}^{{}^{\circ}}$*—*6*d* ^4^P_2½_
8413.59	5*c*	11882.26	*nd* 5_2½_—7*p* ${\;}^{4}D_{2½}^{{}^{\circ}}$
8393.30	1000*cZ*	11910.99	$\{\;\begin{array}{c} 6s{\;}^{4}P_{0½}—6p{\;}^{2}D_{1½}^{{}^{\circ}}\\ 6p{\;}^{2}D_{2½}^{{}^{\circ}}—8s{\;}^{2}P_{1½} \end{array}$
8391.70	200*Z*	11913.27	6*p* ${\;}^{4}S_{1½}^{{}^{\circ}}$*—nd* 21_2½_
8382.49	2*c*	11926.36	*nd* 1_1½_—8*p* ${\;}^{4}P_{1½}^{{}^{\circ}}$
8352.73	40	11968.85	6*p* ${\;}^{4}P_{1½}^{{}^{\circ}}$—*nd* 22_1½_
8333.19	50	11996.91	6*p* ${\;}^{4}P_{2½}^{{}^{\circ}}$—*nd* 21_2½_
8322.16	3*c*	12012.38	*nd* 4_1½_—9*p* ${\;}^{4}P_{2½}^{{}^{\circ}}$
8305.80	40*c*	12036.47	*nd* 3_2½_—9*p* ${\;}^{4}P_{2½}^{{}^{\circ}}$
8289.50	60*d*	12060.14	6*s*′ ^2^D_1½_—6*p*′ ${\;}^{2}P_{1½}^{{}^{\circ}}$
8285.18	8*d*	12066.42	
8279.57	2*d*	12074.21	*nd* 2_1½_—9*p* ${\;}^{4}P_{2½}^{{}^{\circ}}$
8262.54	30	12099.50	
8260.04	200*Z*	12103.15	
8258.84	1	12104.91	*nd* 4.1_1½_—9*p* ${\;}^{4}S_{1½}^{{}^{\circ}}$
8257.74	15	12106.52	
8251.08	80*Z*	12116.30	6*s* ^2^P_0½_—7*p* ${\;}^{4}P_{0½}^{{}^{\circ}}$
8247.74	15*d*	12121.21	
8240.05	4000*Z*	12132.52	*nd* 3_2½_—9*p* ${\;}^{4}S_{1½}^{{}^{\circ}}$
8222.57	500*dZ*	12158.31	6*s* ^4^P_0½_—6*p* ${\;}^{2}P_{1½}^{{}^{\circ}}$
8213.95	50*d*	12171.07	*nd* 2_1½_—9*p* ${\;}^{4}S_{1½}^{{}^{\circ}}$
8206.49	2	12182.28	*nd* 4.1_1½_—9*p* ${\;}^{2}D_{2½}^{{}^{\circ}}$
8187.94	30*c*	12209.73	*nd* 3_2½_—9*p* ${\;}^{2}D_{2½}^{{}^{\circ}}$
8179.00	50*d*	12223.08	6*p* ${\;}^{4}P_{1½}^{{}^{\circ}}$—7*s* ^2^P_0½_
8169.38	800*cZ*	12237.47	6*s*′ ^2^D_1½_*—np* $2_{2½}^{{}^{\circ}}$
8162.22	1	12248.20	*nd* 2_1½_—9*p* ${\;}^{2}D_{2½}^{{}^{\circ}}$
8105.60	50*dZ*	12333.76	6*p* ${\;}^{4}P_{1½}^{{}^{\circ}}$*—nd* 24_1½_
8090.76	1000*Z*	12356.38	6*p* ${\;}^{4}P_{1½}^{{}^{\circ}}$*—nd* 25_2½_
8065.70	300*dZ*	12394.77	6*s*′ ^2^D_2½_—6*p*′ ${\;}^{2}P_{1½}^{{}^{\circ}}$
8046.13	4	12424.75	6*s*′ ^2^D_1½_—7*p* ${\;}^{2}P_{0½}^{{}^{\circ}}$
8043.74	100000*Z*	12428.61	6*s* ^4^P_2½_*—*6*p* ${\;}^{4}P_{1½}^{{}^{\circ}}$
8039.85	100*dZ*	12434.62	6*s* ^2^P_0½_—7p ${\;}^{4}P_{1½}^{{}^{\circ}}$
8023.01	300*Z*	12460.73	$\{\;\begin{array}{c} 6s^{\prime }{\;}^{2}D_{1½}—8p{\;}^{4}S_{1½}^{{}^{\circ}}\\ 6p{\;}^{4}S_{1½}^{{}^{\circ}}—8s{\;}^{4}P_{2½} \end{array}$
8009.61	2	12481.57	
8003.63	1000*Z*	12490.90	6*s* ^4^P_0½_*—*6*p* ${\;}^{4}D_{0½}^{{}^{\circ}}$
7998.01	40	12499.68	6*s*′ ^2^D_1½_—8*p* ${\;}^{2}D_{2½}^{{}^{\circ}}$
7995.63	3	12503.40	
7974.48	200*Z*	12536.56	*nd* 1_1½_—5*f* $1_{1½}^{{}^{\circ}}$
7969.48	500*Z*	12544.42	6*p* ${\;}^{4}P_{2½}^{{}^{\circ}}$—8*s* ^4^P_2½_
7968.61	5	12545.80	*nd* 1_1½_—5*f* $3_{2½}^{{}^{\circ}}$
7960.43	50*d*	12558.68	6*p* ${\;}^{4}P_{0½}^{{}^{\circ}}$*—*7*s* ^4^P_0½_
7955.90	40*dZ*	12565.84	6*p* ${\;}^{4}S_{1½}^{{}^{\circ}}$*—*8*s* ^2^P_1½_
7951.99	200*c*	12572.01	6*s*′ ^2^D_2½_*—np* $2_{1½}^{{}^{\circ}}$
7944.85	250*cZ*	12583.32	*nd* 1_1½_—5*f* $5_{1½}^{{}^{\circ}}$
7941.34	1	12588.87	
7927.94	15*c*	12610.15	
7927.10	50*c*	12611.49	*nd* 4.1_1½_—6*f* $6_{1½}^{{}^{\circ}}$
7922.23	20*c*	12619.24	*nd* 3_2½_—6*f* $2_{3½}^{{}^{\circ}}$
7914.70	15*c*	12631.25	*nd* 3_2½_*—*6*f* $4_{2½}^{{}^{\circ}}$
7909.08	3	12640.22	
7907.86	25	12642.17	
7904.45	10*d*	12647.63	
7903.27	3	12649.51	6*p* ${\;}^{4}P_{2½}^{{}^{\circ}}$*—*8s ^2^P_1½_
7899.41	3*c*	12655.70	*nd* 2_1½_—6*f* $1_{2½}^{{}^{\circ}}$
7897.98	600	12657.99	
7892.43	15*c*	12666.89	*nd* 2_1½_*—*6*f* $3_{1½}^{{}^{\circ}}$
7890.85	25*c*	12669 42	*nd* 2_1½_—6*f* $4_{2½}^{{}^{\circ}}$
7886.00	5*c*	12677.22	
7885.72	5*c*	12677.66	
7864.56	1	12711.77	6*s*′ ^2^D_2½_—8*p* ${\;}^{4}P_{2½}^{{}^{\circ}}$
7861.20	15	12717.21	
7846.21	12*c*	12741.51	
7813.39	50	12795.03	6*s*′ ^2^D_2½_—8*p* ${\;}^{4}S_{1½}^{{}^{\circ}}$
7792.51	4*d*	12829.31	
7789.48	5	12834.30	6*s*′ ^2^D_2½_—8*p* ${\;}^{2}D_{2½}^{{}^{\circ}}$
7778.39	15	12852.60	6*p* ${\;}^{4}P_{1½}^{{}^{\circ}}$—9s ^4^P_2½_
7768.13	25*d*	12869.57	6*s*′ ^2^D_1½_*—*8*p* ${\;}^{4}P_{1½}^{{}^{\circ}}$
7758.80	40*d*	12885.05	6*p* ${\;}^{4}P_{1½}^{{}^{\circ}}$—9s ^2^P_1½_
7728.92	25	12934.86	
7715.72	15*c*	12956.99	*nd* 2_1½_—7*p* ${\;}^{2}S_{0½}^{{}^{\circ}}$
7700.20	2000*cZ*	12983.11	6*s*′ ^2^D_2½_—8*p* ${\;}^{4}D_{3½}^{{}^{\circ}}$
7696.92	50*c*	12988.64	
7671.01	200*dZ*	13032.51	6*p* ${\;}^{4}P_{0½}^{{}^{\circ}}$—*nd* 21.2_0½_
7670.02	10*d*	13034.19	6*p* ${\;}^{4}P_{0½}^{{}^{\circ}}$—7*s* ^4^P_1½_
7611.16	5	13134.99	*nd* 3_2½_—7*p* ${\;}^{4}D_{1½}^{{}^{\circ}}$
7604.88	200*Z*	13145.85	6*s* ^4^P_1½_*—*7*p* ${\;}^{4}P_{2½}^{{}^{\circ}}$
7588.60	400*Z*	13174.04	6*p* ${\;}^{4}P_{0½}^{{}^{\circ}}$*—nd* 22_1½_
7571.25	100	13204.24	6*s*′ ^2^D_2½_*—*8*p* ${\;}^{4}P_{1½}^{{}^{\circ}}$
7556.65	500*dZ*	13229.74	6*s* ^4^P_1½_*—*7*p* ${\;}^{4}S_{1½}^{{}^{\circ}}$
7554.18	2000*Z*	13234.07	6*p* ${\;}^{4}D_{3½}^{{}^{\circ}}$—6*d* ^4^D_3½_
7547.03	5*h*	13246.60	6*p* ${\;}^{2}D_{2½}^{{}^{\circ}}$—7*s* ^4^P_1½_
7539.66	200	13259.55	6*p* ${\;}^{2}D_{2½}^{{}^{\circ}}$—6*d* ^4^D_3½_
7531.81	200*Z*	13273.37	6*p* ${\;}^{4}D_{3½}^{{}^{\circ}}$—6*d* ^4^P_2½_
7490.52	500*cZ*	13346.54	6*s*′ ^2^D_1½_*—*8*p* ${\;}^{2}P_{0½}^{{}^{\circ}}$
7483.97	4	13358.26	
7476.45	25	13371.65	6*s* ^4^P_1½_—7*p* ${\;}^{2}D_{2½}^{{}^{\circ}}$
7468.99	5000*Z*	13385.01	6*p* ${\;}^{4}D_{3½}^{{}^{\circ}}$*—*6*d* ^4^F_4½_
7468.45	10	13385.98	
7468.16	25*d*	13386.50	6*p* ${\;}^{2}D_{2½}^{{}^{\circ}}$—*nd* 22_1½_
7448.11	*2d*	13422.54	*nd* 1_1½_—6*p* ${\;}^{4}S_{1½}^{{}^{\circ}}$
7446.37	300*dZ*	13425.67	6*p* ${\;}^{4}S_{1½}^{{}^{\circ}}$*—*7*s* ^4^P_0½_
7444.91	100*d*	13428.29	6*p* ${\;}^{4}P_{0½}^{{}^{\circ}}$*—*7*s* ^2^P_0½_
7444.08	8	13429.80	
7437.41	8*d*	13441.01	
7421.98	6	13469.78	
7420.01	200	13473.37	
7418.90	12	13475.27	
7418.26	100	13476.54	*nd* 2_1½_—7*f* $1_{2½}^{{}^{\circ}}$
7416.48	500*Z*	13479.78	6*s*′ ^2^D_1½_—5*f* $1_{1½}^{{}^{\circ}}$
7414.50	50	13483.38	6*s* ^4^P_1½_*—*7*p* ${\;}^{4}P_{0½}^{{}^{\circ}}$
7413.60	200*dZ*	13485.01	6*s*′ ^2^D_1½_—5*f* $2_{0½}^{{}^{\circ}}$
7411.20	200*Z*	13489.38	6*s*′ ^2^D_1½_—5*f* $3_{2½}^{{}^{\circ}}$
7410.50	1000*Z*	13490.66	
7402.06	5000*Z*	13506.04	6*p* ${\;}^{2}D_{2½}^{{}^{\circ}}$—*nd* 23_3½_
7390.78	60*c*	13526.65	6*s*′ ^2^D_1½_*—5f* $5_{1½}^{{}^{\circ}}$
7385.58	15*d*	13536.18	
7384.08	150*dZ*	13538.92	6*p* ${\;}^{4}P_{0½}^{{}^{\circ}}$—*nd* 24_1½_
7341.23	20	13617.95	6*p* ${\;}^{4}P_{1½}^{{}^{\circ}}$—7*d* ^4^P_2½_
7336.76	1*cw*	13626.75	*nd* 3_2½_—*np* $6_{2½}^{{}^{\circ}}$
7335.40	3	13628.77	6*p* ${\;}^{4}P_{1½}^{{}^{\circ}}$—*nd* 26_1½_
7333.72	*5cw*	13631.90	6*p* ${\;}^{4}P_{1½}^{{}^{\circ}}$—*nd* 26.1_0½_
7306.15	2	13683.33	
7305.43	400*cZ*	13684.69	6*s*′ ^2^D_1½_—*np* $3_{1½}^{{}^{\circ}}$
7286.44	40	13720.35	6*p* ${\;}^{4}P_{1½}^{{}^{\circ}}$—*nd* 27_1½_
7269 97	25*d*	13751.43	6*p* ${\;}^{2}D_{2½}^{{}^{\circ}}$—*nd* 24_1½_
7263 61	25	13763.48	6*p* ${\;}^{4}P_{1½}^{{}^{\circ}}$—*nd* 27.1_2½_
7259.98	100*cZ*	13770.35	6*s*′ ^2^D_1½_—*np* $4_{2½}^{{}^{\circ}}$
7258.06	200*Z*	13774.00	6*p* ${\;}^{2}D_{2½}^{{}^{\circ}}$—*nd* 25_2½_
7243.49	25	13801.70	6*s* ^4^P_1½_*—*7*p* ${\;}^{4}P_{1½}^{{}^{\circ}}$
7237.84	500*Z*	13812.48	
7236.78	1000*Z*	13814.50	6*s*′ ^2^D_2½_—5*f* $1_{1½}^{{}^{\circ}}$
7235.01	200	13817.88	
7233.81	100	13820.17	6*p* ${\;}^{4}P_{1½}^{{}^{\circ}}$—*nd* 28.1_2½_
7231.82	200*Z*	13823.98	6*s*′ ^2^D_2½_—5*f* $3_{2½}^{{}^{\circ}}$
7230.13	80	13827.21	6*s*′ ^2^D_2½_—5*f* $4_{3½}^{{}^{\circ}}$
7228.94	30	13829.48	
7227.30	700*Z*	13832.62	6*s*′ ^2^D_2½_*—*5*f* $4.1_{3½}^{{}^{\circ}}$
7221.10	5	13844.50	6*s*′ ^2^D_2½_—5*f* $4.2_{3½}^{{}^{\circ}}$
7212.50	20*c*	13861.00	6*s*′ ^2^D_2½_—5*f* $5_{1½}^{{}^{\circ}}$
719Z 52	300*cZ*	13899.51	6*p* ${\;}^{4}S_{1½}^{{}^{\circ}}$—*nd* 21.2_0½_
7191.66	400*dZ*	13901.17	6*p* ${\;}^{4}S_{1½}^{{}^{\circ}}$—7*s* ^4^P_1½_
7182.79	300	13918.34	*nd* 1_1½_—6*f* $3_{2½}^{{}^{\circ}}$
7178.03	30	13927.56	*nd* 1_1½_—6*f* $5_{0½}^{{}^{\circ}}$
7177.35	30*d*	13928.89	*nd* 1_1½_—6*f* $6_{1½}^{{}^{\circ}}$
7164.79	1000*Z*	13953.30	6*p* ${\;}^{4}S_{1½}^{{}^{\circ}}$—6*d* ^4^P_2½_
7148.63	400*cZ*	13984.84	6*p* ${\;}^{4}P_{2½}^{{}^{\circ}}$—7*s* ^4^P_1½_
7142.06	2000*Z*	13997.71	6*p* ${\;}^{4}P_{2½}^{{}^{\circ}}$—6*d* ^4^D_3½_
7137.12	10	14007.39	
7135.55	60	14010.48	*6p* ${\;}^{4}P_{1½}^{{}^{\circ}}$—*nd* 28.2_1½_
7131.06	10*cw*	14019.32	6*s*′ ^2^D_2½_—*np* $3_{1½}^{{}^{\circ}}$
7122.05	1200*Z*	14037.04	6*p* ${\;}^{4}P_{2½}^{{}^{\circ}}$—6*d* ^4^P_2½_
7120.05	500*Z*	14040.98	6*p* ${\;}^{4}S_{1½}^{{}^{\circ}}$—*nd* 22_1½_
7107.43	65*c*	14065.91	*nd* 2_1½_*—np* $7_{1½}^{{}^{\circ}}$
7095.17	80	14090.22	6*p* ${\;}^{4}P_{0½}^{{}^{\circ}}$—9*s* ^2^P_1½_
7087.76	200*c*	14104.95	6*s*′ ^2^D_2½_—*np* $4_{2½}^{{}^{\circ}}$
7085.05	5*c*	14110.34	6*s* ^2^P_0½_—4*f* $0_{1½}^{{}^{\circ}}$
7077.87	300	14124.65	6*p* ${\;}^{4}P_{2½}^{{}^{\circ}}$—*nd* 22_1½_
7063.59	300*Z*	14153.21	6*s* ^4^P_0½_—7*p* ${\;}^{4}S_{1½}^{{}^{\circ}}$
7055.30	20	14169.84	6*s* ^2^P_0½_*—*4*f* $4_{1½}^{{}^{\circ}}$
7045.11	5	14190.34	6*p* ${\;}^{4}P_{1½}^{{}^{\circ}}$—10*s* ^4^P_2½_
7031.62	8*w*	14217.56	6*s* ^2^P_0½_—4*f* $5.1_{1½}^{{}^{\circ}}$
7030.40	5	14220.02	6*s* ^2^P_0½_*—4f* $6_{1½}^{{}^{\circ}}$
7018.42	30	14244.30	*6p* ${\;}^{4}P_{2½}^{{}^{\circ}}$—*nd* 23_3½_
7018.24	100	14244.66	6*p* ${\;}^{4}D_{3½}^{{}^{\circ}}$—9s ^4^P_2½_
7010.20	20	14261.00	
7006.16	5	14269.23	6*s*′ ^2^D_1½_—9*p* ${\;}^{4}P_{2½}^{{}^{\circ}}$
6993.41	100*d*	14295.24	6*p* ${\;}^{4}S_{1½}^{{}^{\circ}}$—7*s* ^2^P_0½_
6991.89	30	14298.34	
6989.78	500	14302.66	6*p* ${\;}^{2}D_{2½}^{{}^{\circ}}$—9*s* ^2^P_1½_
6986.51	400	14309.36	
6985.13	40	14312.18	6*p* ${\;}^{4}P_{1½}^{{}^{\circ}}$—*nd* 29_1½_
6959.09	50	14365.73	6*s*′ ^2^D_1½_—9*p* ${\;}^{4}S_{1½}^{{}^{\circ}}$
6940.98	50*d*	14403.22	
6939.71	8*w*	14405.86	6*p* ${\;}^{4}S_{1½}^{{}^{\circ}}$—*nd* 24_1½_
6939.21	15*c*	14406.90	6*s* ^4^P_0½_—7*p* ${\;}^{4}P_{0½}^{{}^{\circ}}$
6928.82	100	14428.50	6*p* ${\;}^{4}S_{1½}^{{}^{\circ}}$—*nd* 25_2½_
6922.05	2	14442.62	6*s*′ ^3^D_1½_—9*p* ${\;}^{2}D_{2½}^{{}^{\circ}}$
6899.61	20*d*	14489.58	6*p* ${\;}^{4}P_{2½}^{{}^{\circ}}$—*nd* 24_1½_
6856.75	50	14580.15	6*p* ${\;}^{4}P_{1½}^{{}^{\circ}}$—*nd* 31_1½_
6849.46	10	14595.66	
6845.63	40	14603.84	$\{\;\begin{array}{c} 6p{\;}^{4}P_{1½}^{{}^{\circ}}—nd\;30_{0½}\\ 6s^{\prime }{\;}^{2}D_{2½}—9p{\;}^{4}P_{2½}^{{}^{\circ}} \end{array}$
6843.07	15	14609.29	
6832.*42*	1	14632.07	6*p* ${\;}^{4}P_{1½}^{{}^{\circ}}$—*nd* 31.1_1½_
6831.56	3	14633.90	6*p* ${\;}^{4}P_{1½}^{{}^{\circ}}$*—*8*d* ^4^P_2½_
6812.30	50	14675.29	6*p* ${\;}^{4}P_{1½}^{{}^{\circ}}$—*nd* 32_1½_
6808.85	10	14682.71	6*p* ${\;}^{4}P_{1½}^{{}^{\circ}}$—*nd* 32.1_0½_
6789.23	60*Z*	14725.15	6*s* ^4^P_0½_—7*p* ${\;}^{4}P_{1½}^{{}^{\circ}}$
6788.04	20	14727.72	*nd* 1_1½_—7*f* $1_{1½}^{{}^{\circ}}$
6784.58	50	14735.25	*nd* 1_1½_—7*p* ${\;}^{4}D_{2½}^{{}^{\circ}}$
6765.27	2	14777.30	6*s*′ ^2^D_2½_*—*9*p* ${\;}^{2}D_{2½}^{{}^{\circ}}$
6741.52	300*c*	14829.36	
6739.44	100	14833.94	6*p* ${\;}^{4}P_{0½}^{{}^{\circ}}$—*nd* 26_1½_
6738.05	100*d*	14837.00	6*p* ${\;}^{4}P_{0½}^{{}^{\circ}}$—*nd* 26.1_0½_
6736.53	100	14840.35	
6732.03	400*Z*	14850.27	6*s*′ ^2^D_1½_*—*6*f* $1_{2½}^{{}^{\circ}}$
6726.92	200*cZ*	14861.55	6*s*′ ^2^D_1½_—6*f* $3_{1½}^{{}^{\circ}}$
6722.73	20*cw*	14870.81	6*s*′ ^2^D_1½_—6*f* $5_{0½}^{{}^{\circ}}$
6722.12	8*cw*	14872.15	6*s*′ ^2^D_1½_—6*f* $6_{1½}^{{}^{\circ}}$
6702.35	5*c*	14916.02	*nd* 1_1½_—*np* 6*f* $6_{2½}^{{}^{\circ}}$
6698.56	25*Z*	14924.47	6*s*′ ^2^D_2½_—9*p* ${\;}^{4}D_{3½}^{{}^{\circ}}$
6698.46	200*Z*	14924.69	6*p* ${\;}^{4}S_{1½}^{{}^{\circ}}$—9*s* ^4^P_2½_
6698.10	60	14925.49	6*p* ${\;}^{4}P_{0½}^{{}^{\circ}}$—*nd* 27_1½_
6697.29	500*cZ*	14927.30	
6683.92	50	14957.16	6*p* ${\;}^{4}S_{1½}^{{}^{\circ}}$—9*s* ^2^P_1½_
6662.10	400*Z*	15006.15	6*p* ${\;}^{4}D_{3½}^{{}^{\circ}}$—7*d* ^4^D_3½_
6661.11	500*Z*	15008.38	6*p* ${\;}^{4}P_{2½}^{{}^{\circ}}$—9*s* ^4^P_2½_
6660.34	100	15010.11	6*p* ${\;}^{4}D_{3½}^{{}^{\circ}}$—7*d* ^4^P_2½_
6650.79	50	15031.66	$\{\;\begin{array}{c} 6p{\;}^{2}D_{2½}^{{}^{\circ}}—7d{\;}^{4}D_{3½}\\ 6p{\;}^{4}P_{1½}^{{}^{\circ}}—nd\;33_{0½} \end{array}$
6644.26	30	15046.43	6*p* ${\;}^{2}D_{2½}^{{}^{\circ}}$—*nd* 26_1½_
6619.66	5000*Z*	15102.35	6*p* ${\;}^{4}D_{3½}^{{}^{\circ}}$—7*d* ^4^F_4½_
6604.07	18*c*	15138.00	6*p* ${\;}^{2}D_{2½}^{{}^{\circ}}$—*nd* 27_1½_
6598.08	12*c*	15151.74	6*s*′ ^2^D_1½_—7*p* ${\;}^{2}S_{0½}^{{}^{\circ}}$
6588.67	4	15173.38	6*p* ${\;}^{4}P_{1½}^{{}^{\circ}}$—*nd* 35_0½_
6585.27	1000*Z*	15181.22	6*p* ${\;}^{2}D_{2½}^{{}^{\circ}}$—*nd* 27.1_2½_
6583.75	2000*Z*	15184.73	6*s*′ ^2^D_2½_—6*f* $1_{2½}^{{}^{\circ}}$
6582.92	300	15186.64	6*s*′ ^2^D_2½_—6*f* $2_{3½}^{{}^{\circ}}$
6581.30	25	15190.37	
6580.53	200	15192.16	
6578.78	50	15196.20	6*s*′ ^2^D_2½_—6*f* $3_{1½}^{{}^{\circ}}$
6577.68	100*d*	15198.74	$\{\;\begin{array}{c} 6s^{\prime }{\;}^{2}D_{2½}—6f\;4_{2½}^{{}^{\circ}}\\ 6p{\;}^{4}D_{3½}^{{}^{\circ}}—nd\;28_{3½} \end{array}$
6575.35	80	15204.13	
6574.21	20*cw*	15206.75	6*s*′ ^2^D_2½_—6*f* $6_{1½}^{{}^{\circ}}$
6570.38	150*Z*	15215.62	6*p* ${\;}^{4}P_{0½}^{{}^{\circ}}$—*nd* 28.2_1½_
6566.49	1000*Z*	15224.64	6*p* ${\;}^{2}D_{2½}^{{}^{\circ}}$—nd 28_3½_
6564.80	200*cZ*	15228.56	6*s* ${\;}^{2}P_{0½}$—6p′ ${\;}^{2}P_{1½}^{{}^{\circ}}$
6560.82	300*Z*	15237.79	6*p* ${\;}^{2}D_{2½}^{{}^{\circ}}$—nd 28.1_2½_
6547.34	50	15269.17	
6489.11	6	15406.18	6*s* ^2^P_0½_*—np* $2_{1½}^{{}^{\circ}}$
6488.10	300*Z*	15408.58	6*s* ^2^P_1½_—6*p* ${\;}^{2}P_{0½}^{{}^{\circ}}$
6479.89	60	15428.11	6*p* ${\;}^{2}D_{2½}^{{}^{\circ}}$*—nd* 28.2_1½_
6479.24	50	15429.65	6*p* ${\;}^{4}P_{1½}^{{}^{\circ}}$*—nd* 37_2½_
6477.39	20	15434.06	6*p* ${\;}^{4}P_{1½}^{{}^{\circ}}$*—nd* 38_1½_
6455.00	50*Z*	15487.60	6*s* ^4^P_1½_—4*f* $1_{2½}^{{}^{\circ}}$
6442.58	30	15517.45	6*p* ${\;}^{4}P_{0½}^{{}^{\circ}}$*—nd* 29_1½_
6434.49	40*Z*	15536.96	6*s* ^4^P_1½_—4*f* $4_{1½}^{{}^{\circ}}$
6433.28	30*Z*	15539.88	6*s* ^4^P_1½_—4*f* $5_{2½}^{{}^{\circ}}$
6415.70	100	15582.46	
6414.80	12*d*	15584.64	6*s* ^4^P_1½_—4*f* $5.1_{1½}^{{}^{\circ}}$
6413.84	12*d*	15586.98	6*s* ^4^P_1½_—4*f* $6_{1½}^{{}^{\circ}}$
6411.22	50*cZ*	15593.35	6*s* ^2^P_0½_—7*p* ${\;}^{2}P_{0½}^{{}^{\circ}}$
6378.70	200	15672.85	6*s*′ ^2^D_2½_—*np* $5_{3½}^{{}^{\circ}}$
6376.33	80*d*	15678.68	6*s*′ ^2^D_1½_*—*7*p* ${\;}^{4}D_{2½}^{{}^{\circ}}$
6371.68	400*Z*	15690.12	6*p* ${\;}^{4}S_{1½}^{{}^{\circ}}$*—*7*d* ^4^P_2½_
6367.28	400*dZ*	15700.96	6*p* ${\;}^{4}S_{1½}^{{}^{\circ}}$*—nd* 26_1½_
6366.67	5*c*	15702.47	6*s*′ ^2^D_2½_*—*7*p* ${\;}^{4}D_{1½}^{{}^{\circ}}$
6366.05	10*cw*	15703.98	
6359.16	500*Z*	15721.31	6*s* ^2^P_1½_*—*6*p* ${\;}^{2}S_{0½}^{{}^{\circ}}$
6355.57	150	15729.89	6*p* ${\;}^{2}D_{2½}^{{}^{\circ}}$*—nd* 29_1½_
6339.44	1000*Z*	15769.91	6*p* ${\;}^{4}P_{2½}^{{}^{\circ}}$—7*d* ^4^D_3½_
6337.85	2000*Z*	15773.87	6*p* ${\;}^{4}P_{2½}^{{}^{\circ}}$*—*7*d* ^4^P_2½_
6333.50	400	15784.70	6*p* ${\;}^{4}P_{2½}^{{}^{\circ}}$—*nd* 26_1½_
6330.37	800*Z*	15792.51	6*p* ${\;}^{4}S_{1½}^{{}^{\circ}}$*—nd* 27_1½_
6323.82	70	15808.86	6*p* ${\;}^{4}P_{0½}^{{}^{\circ}}$*—nd* 30_0½_
6313.13	500*Z*	15835.63	6*p* ${\;}^{4}S_{1½}^{{}^{\circ}}$—*nd* 27.1_2½_
6312.50	100	15837.21	6*p* ${\;}^{4}P_{0½}^{{}^{\circ}}$*—nd* 31.1_1__½_
6303.93	1	15858.74	6*s*′ ^2^D_1__½_—*np* $6_{2½}^{{}^{\circ}}$
6297.00	100*dZ*	15876.20	6*p* ${\;}^{4}P_{2½}^{{}^{\circ}}$*—nd* 27_1__½_
6295.24	30	15880.63	6*p* ${\;}^{4}P_{0½}^{{}^{\circ}}$*—nd* 32_1__½_
6293.98	1000*Z*	15883.81	6*s* ^2^P_1__½_—6*p* ${\;}^{4}D_{1½}^{{}^{\circ}}$
6292.36	10*cw*, Iii?	15887.90	6*p* ${\;}^{4}P_{0½}^{{}^{\circ}}$—*nd* 32.1_0½_
6290.61	100	15892.32	6*p* ${\;}^{4}S_{1½}^{{}^{\circ}}$—*nd* 28.1_2½_
6280.03	2*w*	15919.10	6*p* ${\;}^{4}P_{2½}^{{}^{\circ}}$*—nd* 27.1_2½_
6262.78	40	15962.94	6*p* ${\;}^{4}P_{2½}^{{}^{\circ}}$—*nd* 28_3½_
6259.12	25	15972.27	
6249.14	40	15997.78	6*p* ${\;}^{2}D_{2½}^{{}^{\circ}}$—*nd* 31_1½_
6246.14	200	16005.53	6*s*′ ^2^D_2½_—7*f* $1_{2½}^{{}^{\circ}}$
6245.38	80	16007.42	
6244.72	40	16009.10	
6244.48	800*Z*	16009.72	6*s*′ ^2^D_2½_—7*f* $2_{3½}^{{}^{\circ}}$
6244.00	100	16010.96	
6243.17	50*d*	16013.08	6*s*′ ^2^D_2½_—7*p* ${\;}^{4}D_{2½}^{{}^{\circ}}$
6242.70	10	16014.29	6*s*′ ^2^D_2½_—10*p* ${\;}^{4}D_{3½}^{{}^{\circ}}$
6240.83	200	16019.09	6*p* ${\;}^{4}D_{3½}^{{}^{\circ}}$—8*d* ^4^D_3½_
6238.12	8	16026.04	6*p* ${\;}^{4}D_{3½}^{{}^{\circ}}$ ^4^P_2½_
6233.50	100*Z*	16037.92	6*s* ^2^P_0½_—8*p* ${\;}^{4}P_{1½}^{{}^{\circ}}$
6230.91	20	16044.58	6*p* ${\;}^{2}D_{2½}^{{}^{\circ}}$—8*d* ^4^D_3½_
6228.91	4*h*	16049.73	6*p* ${\;}^{2}D_{2½}^{{}^{\circ}}$—*nd* 31.1_1__½_
6228.20	40	16051.57	6*p* ${\;}^{2}D_{2½}^{{}^{\circ}}$—8*d* ^4^P_2½_
6216.17	1*c*	16082.62	6*p* ${\;}^{4}S_{1½}^{{}^{\circ}}$—*nd* 28.2_1__½_
6213.10	500*Z*	16090.58	6*p* ${\;}^{4}D_{3½}^{{}^{\circ}}$—8*d* ^4^F_4½_
6212.14	3*c*	16093.06	6*p* ${\;}^{2}D_{2½}^{{}^{\circ}}$—*nd* 32_1__½_
6191.88	800*Z*	16145.73	
6183.98	5*c*	16166.34	6*p* ${\;}^{4}P_{2½}^{{}^{\circ}}$—*nd* 28.2_1__½_
6173.62	3*h*	16193.48	6*s*′ ^2^D_2½_—*np* $6_{2½}^{{}^{\circ}}$
6168.71	3	16206.37	
6147.43	50	16262.47	6*p* ${\;}^{4}S_{1½}^{{}^{\circ}}$—10*s* ^4^P_2½_
6115.97	100*Z*	16346.12	6*p* ${\;}^{4}P_{2½}^{{}^{\circ}}$—10*s* ^4^P_2½_
6101.71	20	16384.31	
6082.43	1000*Z*	16436.26	6*s* ^2^P_1½_—6*p* ${\;}^{4}D_{2½}^{{}^{\circ}}$
6073.46	50*Z*	16460.53	6*s* ^4^P_0½_—4*f* $4_{1½}^{{}^{\circ}}$
6055.96	80*Z*	16508.10	6*S* ^4^P_0½_*—*4*f* $5.1_{1½}^{{}^{\circ}}$
6055.03	30*d*	16510.63	6*s* ^4^P_0½_—4*f* $6_{1½}^{{}^{\circ}}$
6053.49	300*dZ*	16514.83	6*s* ^2^P_0½_—8*p* ${\;}^{2}P_{0½}^{{}^{\circ}}$
6044.41	60	16539.64	
6042.71	100	16544.30	
6041.24	10	16548.31	
6024.08	2000*dZ*	16595.46	$\left\{ \begin{array}{l} \;6s\prime {\;}^{2}D_{2½}—np\;7_{1½}^{{}^{\circ}}\\ \;6s{\;}^{4}P_{1½}—6p\prime {\;}^{2}P_{1½}^{{}^{\circ}} \end{array}\right.$
6015.37	40	16619.49	6*p* ${\;}^{4}D_{3½}^{{}^{\circ}}$—9*d* ^4^D_3½_
6004.99	20	16648.22	6*s* ^2^P_0½_—5*f* $1_{1½}^{{}^{\circ}}$
6003.54	3	16652.23	6*p* ${\;}^{4}S_{1½}^{{}^{\circ}}$*—nd* 31_1½_
5984.86	300	16704.21	$\left\{ \begin{array}{l} \;6p{\;}^{4}D_{3½}^{{}^{\circ}}—9d{\;}^{4}F_{4½}\\ \;6p{\;}^{4}S_{1½}^{{}^{\circ}}—nd\;31.1_{1½} \end{array}\right.$
5984.23	200	16705.97	6*p* ${\;}^{4}S_{1½}^{{}^{\circ}}$—8*d* ^4^P_2½_
5981.26	40*Z*	16714.26	6*s* ^2^P_1½_—6*p* ${\;}^{2}D_{1½}^{{}^{\circ}}$
5980.84	100	16715.44	6*s* ^4^P_1½_—*np* $1.1_{2½}^{{}^{\circ}}$
5976.53	12	16727.48	6*p* ${\;}^{4}D_{3½}^{{}^{\circ}}$*—nd* 36_2½_
5973.50	60	16735.98	6*p* ${\;}^{4}P_{2½}^{{}^{\circ}}$*—nd* 31_1½_
5969.36	1	16747.58	6*p* ${\;}^{4}S_{1½}^{{}^{\circ}}$—*nd* 32_1½_
5968.26	150	16750.67	
5967.81	4	16751.92	6*p* ${\;}^{4}P_{0½}^{{}^{\circ}}$—*nd* 38.2_1½_
5967.46	3	16752.92	6*p* ${\;}^{2}D_{2½}^{{}^{\circ}}$—*nd* 36_2½_
5966.76	100	16754.88	6*p* ${\;}^{4}S_{1½}^{{}^{\circ}}$*—nd* 32.1_0½_
5960.40	300*cZ*	16772.76	6*s* ^4^P_1½_—*np* $2_{1½}^{{}^{\circ}}$
5956.87	300	16782.70	$\left\{ \begin{array}{l} \;6p{\;}^{4}P_{2½}^{{}^{\circ}}—8d{\;}^{4}D_{3½}\\ \;6s{\;}^{4}P_{1½}—np\;1_{1½}^{{}^{\circ}} \end{array}\right.$
5955.00	50	16787.96	
5954.38	150	16789.72	6*p* ${\;}^{4}P_{2½}^{{}^{\circ}}$—8*d* ^4^P_2½_
5947.78	2	16808.34	
5943.07	1	16821.67	6*p* ${\;}^{4}D_{3½}^{{}^{\circ}}$—*nd* 37_2½_
5934.03	25	16847.29	6*p* ${\;}^{2}D_{2½}^{{}^{\circ}}$—*nd* 37_2½_
5932.02	8*cw*	16853.00	6*s* ^2^P_0½_—*np* $3_{1½}^{{}^{\circ}}$
5911.17	20	16912.45	6*s* ^4^P_1½_—8*p* ${\;}^{4}P_{2½}^{{}^{\circ}}$
5894 03	2000*Z*	16961.63	6*s* ^2^P_1½_—6*p* ${\;}^{2}P_{1½}^{{}^{\circ}}$
5882.24	70*Z*	16995.62	6*s* ^4^P_1½_—8*p* ${\;}^{4}S_{1½}^{{}^{\circ}}$
5868.68	3	17034.90	6*s* ^4^P_1½_—8*p* ${\;}^{2}D_{2½}^{{}^{\circ}}$
5845.04	6	17103.98	6*p* ${\;}^{4}S_{1½}^{{}^{\circ}}$*—nd* 33_0__½_
5829.85	50	17148.36	6*p* ${\;}^{4}D_{3½}^{{}^{\circ}}$—10*d* ^4^F_4½_
5823.20	50	17167.94	6*p* ${\;}^{4}P_{2½}^{{}^{\circ}}$—11*s* ^4^P_2½_
5810.19	5*h*	17206.37	6*p* ${\;}^{2}D_{2½}^{{}^{\circ}}$—*nd* 40_2½_
5780.65	80*Z*	17294.31	6*s* ^2^P_1½_*—*6*p* ${\;}^{4}D_{0½}^{{}^{\circ}}$
5764.33	1000*dZ*	17343.27	6*s* ^4^P_2½_—6*p* ${\;}^{4}D_{1½}^{{}^{\circ}}$
5751.06	100	17383.29	$\left\{ \begin{array}{l} \;6p{\;}^{4}P_{2½}^{{}^{\circ}}—9d{\;}^{4}D_{3½}\\ \;6s{\;}^{4}P_{1½}—8p\;{\;}^{4}P_{0½}^{{}^{\circ}} \end{array}\right.$
5743.90	150*Z*	17404.96	6*s* ^4^P_1½_—8*p* ${\;}^{4}P_{1½}^{{}^{\circ}}$
5742.98	30	17407.74	6*p* ${\;}^{4}S_{1½}^{{}^{\circ}}$—*nd* 36_2½_
5732.66	6*cw*	17439.07	
5715.55	40	17491.29	6*p* ${\;}^{4}P_{2½}^{{}^{\circ}}$*—nd* 36_2½_
5706.05	5	17520.40	
5684.92	5	17585.52	6*p* ${\;}^{4}P_{2½}^{{}^{\circ}}$—*nd* 37_2½_
5683.48	4	17589.98	
5682.00	5	17594.55	6*p* ${\;}^{4}S_{1½}^{{}^{\circ}}$*—nd* 38.1_2½_
5674.14	2	17618.93	6*p* ${\;}^{4}S_{1½}^{{}^{\circ}}$—*nd* 38.2_1½_
5637.00	3	17735.01	6*p* ${\;}^{4}P_{2½}^{{}^{\circ}}$*—nd* 39_2½_
5597.28	2	17860.87	6*p* ${\;}^{4}S_{1½}^{{}^{\circ}}$*—nd* 40^2^_½_
5590.65	30*cw*	17882.05	6*s* ^4^P_1½_—8*p* ${\;}_{2}P_{0½}^{\;{}^{\circ}}$
5590.20	*8cw*	17883.50	6*s* ^4^P_0½_—7*r* ${\;}^{2}P_{0½}^{{}^{\circ}}$
5586.36	400*cZ*	17895.79	6*s* ^4^P_2½_—6*p* ${\;}^{4}D_{2½}^{{}^{\circ}}$
5579.05	40	17919.24	6*s* ^4^P_0½_—8*p* ${\;}^{4}S_{1½}^{{}^{\circ}}$
5549.32	25	18015.24	6*s* ^4^P_1½_—5*f* $1_{1½}^{{}^{\circ}}$
5546.41	70	18024.69	6*s* ^4^P_1½_—5*f* $3_{2½}^{{}^{\circ}}$
5544.80	1	18029.92	6*s* ^2^P_0½_—6*f* $3_{1½}^{{}^{\circ}}$
5536.03	1	18058.47	6*s* ${\;}^{4}S_{1½}^{{}^{\circ}}$—6*s″* ${\;}^{2}S_{0½}$
5534.98	10*h*	18061.91	6*s* ^4^P_1½_—5*f* $5_{1½}^{{}^{\circ}}$
5500.95	150*cZ*	18173.64	6*s* ^4^P_2½_—6*p* ${\;}^{2}D_{1½}^{{}^{\circ}}$
5486.94	3*d*	18220.03	6*s* ^4^P_1½_—*np* $3_{1½}^{{}^{\circ}}$
5461.24	7*hl*	18305.79	6*s* ^4^P_1½_—*np* $4_{2½}^{{}^{\circ}}$
5427.94	10	18418.08	
5427.06	600*c*	18421.08	6*s* ^4^P_2½_—6*p* ${\;}^{2}P_{1½}^{{}^{\circ}}$
5316.36	1	18804.64	6*s* ^4^P_1½_—9*p* ${\;}^{4}P_{2½}^{{}^{\circ}}$
5300.99	25	18859.15	
5297.17	20*Z*	18872.77	6*s* ^2^P_1½_—7*p* ${\;}^{4}P_{1½}^{{}^{\circ}}$
5278.73	10	18938.69	6*s* ^4^P_0½_—5*f* $1_{1½}^{{}^{\circ}}$
5273.72	30*Z*	18956.69	6*s* ^2^P_1½_—7*p* ${\;}^{4}S_{1½}^{{}^{\circ}}$
5265.69	40*Z*	18985.59	6*s* ^4^P_0½_—5*f* $5_{1½}^{{}^{\circ}}$
5238.26	20	19085.01	
5234.57	1000*Z*	19098.46	6*s* ^2^P_1½_—7*p* ${\;}^{2}D_{2½}^{{}^{\circ}}$
5204.15	300*Z*	19210.10	6*s* ^2^P_1½_—7*p* ${\;}^{4}P_{0½}^{{}^{\circ}}$
5196.77	10	19237.38	6*s* ^2^P_0½_—7*p* ${\;}^{2}D_{1½}^{{}^{\circ}}$
5194.86	1	19244.44	6*s* ^2^P_0½_—7*p* ${\;}^{2}P_{1½}^{{}^{\circ}}$
5154.03	3	19396.90	6*s* ^4^P_1½_—6*f* $3_{1½}^{{}^{\circ}}$
5153.37	3	19399.39	6*s* ^4^P_1½_—6*f* $4_{2½}^{{}^{\circ}}$
5145.52	400*Z*	19428.98	6*s* ^2^P_0½_—*np* $7_{1½}^{{}^{\circ}}$
5119.29	10000*Z*	19528.53	6*s* ^2^P_1½_—7*p* ${\;}^{4}P_{1½}^{{}^{\circ}}$
5029.34	4	19877.58	6*s* ^4^P_1½_*—*10*p* ${\;}^{4}P_{2½}^{{}^{\circ}}$
4947.59	15	20206.24	6*s* ^4^P_1½_—7*f* $1_{2½}^{{}^{\circ}}$
4945.74	7	20213.79	6*s* ^4^P_1½_—7*p* ${\;}^{4}D_{2½}^{{}^{\circ}}$
4919.80	3	20320.37	6*s* ^4^P_0½_—6*f* $3_{1½}^{{}^{\circ}}$
4916.94	200*Z*	20332.19	6*s* ^4^P_2½_—7*p* ${\;}^{4}P_{2½}^{{}^{\circ}}$
4902.00	75*Z*	20394.16	6*s* ^4^P_1½_—*np* $6_{2½}^{{}^{\circ}}$
4896.75	200	20416.02	6*s* ^4^P_2½_*—*7*p* ${\;}^{4}S_{1½}^{{}^{\circ}}$
4882.68	12	20474.86	
4874.55	2	20509.00	
4862.96	60*Z*	20557.88	6*s* ^4^P_2½_—7*p* ${\;}^{2}D_{2½}^{{}^{\circ}}$
4862.32	1000*Z*	20560.59	6*s* ^4^P_2½_—7*p* ${\;}^{4}D_{3½}^{{}^{\circ}}$
4850.51	60Z	20610.65	6*s* ^4^P_0½_—7*p* ${\;}^{2}S_{0½}^{{}^{\circ}}$
4850.35	50*Z*	20611.37	6*s* ^4^P_1½_—7*p* ${\;}^{2}P_{1½}^{{}^{\circ}}$
4827.57	35*Z*	20708.59	
4802.46	5	20816.86	
4800.20	50	20826.66	6*s* ^4^P_0½_—7*p* ${\;}^{4}D_{1½}^{{}^{\circ}}$
4763.31	250*Z*	20987.95	6*s* ^4^P_2½_*—*7*p* ${\;}^{4}P_{1½}^{{}^{\circ}}$
4701.52	5	21263.79	6*s* ^2^P_1½_*—*4*f* $4_{1½}^{{}^{\circ}}$
4700.88	40*Z*	21266.68	6*s* ^2^P_1½_—4*f* $5_{2½}^{{}^{\circ}}$
4690.90	20*c*	21311.92	6*s* ^2^P_1½_—4*f* $5.1_{1½}^{{}^{\circ}}$
4690.49	35*Z*	21313.79	6*s* ^2^P_1½_—4*f* $6_{1½}^{{}^{\circ}}$
4643.84	1	21527.89	6*s* ^4^P_0½_—7*p* ${\;}^{2}D_{1½}^{{}^{\circ}}$
4642.32	8	21534.94	6*s* ^4^P_0½_—7*p* ${\;}^{2}P_{1½}^{{}^{\circ}}$
4602.86	12	21719.56	6*s* ^4^P_0½_—*np* $7_{1½}^{{}^{\circ}}$
4478.56	100*Z*	22322.36	6*s* ^2^P_1½_—6*p*′ ${\;}^{2}P_{1½}^{{}^{\circ}}$
4443.26	15*d*	22499.70	6s ^2^P_1½_—*np* $2_{2½}^{{}^{\circ}}$
4409.12	15*Z*	22673.92	6*s* ^4^P_2½_—4*f* $1_{2½}^{{}^{\circ}}$
4408.95	20	22674.78	
4407.94	25*Z*	22679.96	6*s* ^4^P_2½_—4*f* $2_{3½}^{{}^{\circ}}$
4407.86	20*c*, *Z*	22680.38	6*s* ^4^P_2½_—4*f* $2.1_{3½}^{{}^{\circ}}$
4406.54	10*Z*	22687.19	$\left\{ \begin{array}{l} 6s{\;}^{2}P_{1½}—7p{\;}^{2}P_{3½}^{{}^{\circ}}\\ 6s{\;}^{4}P_{2½}—4f\;3_{3½}^{{}^{\circ}} \end{array}\right.$
4398.99	40*cZ*	22726.13	6*s* ^4^P_2½_—4*f* $5_{2½}^{{}^{\circ}}$
4392.09	40*Z*	22761.83	6*s* ^2^P_1½_—8*p* ${\;}^{2}D_{2½}^{{}^{\circ}}$
4389.85	5*c*	22773.44	6s ^4^P_2½_—4*f* $6_{1½}^{{}^{\circ}}$
4321.84	500*dZ*	23131.81	6*s* ^2^P_1½_—8*p* ${\;}^{4}P_{1½}^{{}^{\circ}}$
4318.36	3	23150.45	
4317.52	2	23154.95	
4292.36	3*d*	23290.68	
4282.75	10*d*	23342.94	
4273.36	4	23394.23	
4265.33	2	23438.27	
4234.54	75*dZ*	23608.69	6*s* ^2^P_1½_—8*p* ${\;}^{2}P_{0½}^{{}^{\circ}}$
4209.82	30*Z*	23747.32	6*s* ^2^P_1½_—5*f* $2_{0½}^{{}^{\circ}}$
4209.06	20	23751.60	6*s* ^2^P_1½_—5*f* $3_{2½}^{{}^{\circ}}$
4203.72	35*dZ*	23781.78	6*s* ^4^P_2½_—6*p*′ ${\;}^{2}P_{1½}^{{}^{\circ}}$
4202.51	8*d*	23788.62	6*s* ^2^P_1½_—5*f* $5_{1½}^{{}^{\circ}}$
4189.16	4	23864.43	
4174.70	1	23947.09	6*s* ^2^P_1½_—*np* $3_{1½}^{{}^{\circ}}$
4172.61	5*w*	23959.08	6*s* ^4^P_2½_—*np* $2_{2½}^{{}^{\circ}}$
4159.85	25*c*	24032.58	6*s* ^2^P_1½_—*np* $4_{2½}^{{}^{\circ}}$
4148.41	75*dZ*	24098.85	6*s* ^4^P_2½_—8*p* ${\;}^{4}P_{2½}^{{}^{\circ}}$
4134.15	100*dZ*	24181.97	6*s* ^4^P_2½_—8*p* ${\;}^{4}S_{1½}^{{}^{\circ}}$
4129.21	200*Z*	24210.90	
4127.43	15	24221.34	6*s* ^4^P_2½_—8*p* ${\;}^{4}D_{2½}^{{}^{\circ}}$
4125.08	3*w*	24235.14	
4102.23	200	24370.13	6*s* ^4^P_2½_—8*p* ${\;}^{4}D_{3½}^{{}^{\circ}}$
4069.48	8	24566.25	
4065.33	40*d*	24591.33	6*s* ^4^P_2½_—8*p* ${\;}^{4}P_{1½}^{{}^{\circ}}$
4059.27	15	24628.04	6*s* ^2^P_1½_—9*p* ${\;}^{4}S_{1½}^{{}^{\circ}}$
4046.63	80	24704.97	6*s* ^2^P_1½_—9*p* ${\;}^{2}D_{2½}^{{}^{\circ}}$
3990.82	2*w*	25050.45	
3978.76	10	25126.38	6*s* ^2^P_1½_—6*f* $4_{2½}^{{}^{\circ}}$
3977.52	5*w*	25134.21	6*s* ^2^P_1½_—6*f* $6_{1½}^{{}^{\circ}}$
3964.89	20*c*	25214.27	6*s* ^4^P_2½_—5*f* $4_{3½}^{{}^{\circ}}$
3934.91	20	25406.37	6*s* ^4^P_2½_—*np* $3_{1½}^{{}^{\circ}}$
3933.73	2	25413.93	6*s* ^2^P_1½_—7*p* ${\;}^{2}S_{0½}^{{}^{\circ}}$
3921.68	55*d*	25492.08	6*s* ^4^P_2½_—*np* $4_{2½}^{{}^{\circ}}$
3918.60	6	25512.12	
3902.02	6	25620.52	
3900.55	8	25630.17	6*s* ^2^P_1½_—7*p* ${\;}^{4}D_{1½}^{{}^{\circ}}$
3893.84	20	25674.34	
3853.86	4	25940.68	6*s* ^2^P_1½_—7*p* ${\;}^{4}D_{2½}^{{}^{\circ}}$
3847.63	15	25982.68	
3846.41	25*d*	25990.92	6*s* ^4^P_2½_—9*p* ${\;}^{4}P_{2½}^{{}^{\circ}}$
3840.95	50	26027.87	
3827.24	20	26121.11	6*s* ^2^P_1½_—*np* $6_{2½}^{{}^{\circ}}$
3820.91	25*d*	26164.38	6*s* ^4^P_2½_—9*p* ${\;}^{2}D_{2½}^{{}^{\circ}}$
3796.69	15	26331.29	6*s* ^2^P_1½_—7*p* ${\;}^{2}D_{1½}^{{}^{\circ}}$
3762.04	6*w*	26573.80	6*s* ^4^P_2½_—6*f* $2_{3½}^{{}^{\circ}}$
3760.34	3*w*	26585.81	6*s* ^4^P_2½_—6*f* $4_{2½}^{{}^{\circ}}$
3698.10	*2w*	27033.25	
3694.42	1	27060.17	6*s* ^4^P_2½_—*np* $5_{3½}^{{}^{\circ}}$
3693.90	20*d*	27063.98	6*s* ^4^P_2½_—10*p* ${\;}^{4}P_{2½}^{{}^{\circ}}$
3690.42	8*d*	27089.50	6*s* ^4^P_2½_—7*p* ${\;}^{4}D_{1½}^{{}^{\circ}}$
3684.42	5	27133.62	
3648.59	1*w*	27400.07	6*s* ^4^P_2½_—7*p* ${\;}^{4}D_{2½}^{{}^{\circ}}$
3624.72	5*c*	27580.50	6*s* ^4^P_2½_*—np* $6_{2½}^{{}^{\circ}}$
3607.55	5*w*	27711.77	
3597.29	3*w*	27790.80	6*s* ^4^P_2½_—7*p* ${\;}^{2}D_{1½}^{{}^{\circ}}$
3596.39	1*w*	27797.76	6s ^4^P_2½_—7*p* ${\;}^{2}P_{1½}^{{}^{\circ}}$
3552.78	3*w*	28138.96	
2061.633	2000	48489.73	5*p*^5^ ${\;}^{2}P_{0½}^{{}^{\circ}}$—6*s* ^2^P_1½_

**Table 2:** Infrared lines of I i

Computed wave length	Intensity	Wave number	Designation

23070.01	95	4333.45	5*d* ^4^D_3½_—5*f* 3212o
23001.54	100	4346.35	7*p* 4S112o—*nd* 24_1½_
22308.77	150	4481.32	6*p* 4D312o—*nd* 5_2½_
22226.19	250	4497.97	6*p* 4P012o*—nd* 5.1_1½_
22182.49	375	4506.83	6*p* 2D212o—*nd* 5_2½_
21569.51	110	4634.91	6*p* 2P012o—*nd* 19_1½_
20153.00	30	4960.69	6*s* ^4^P_0½_—6p 4P012o
19910.52	45	5021.10	*nd* 13_2½_—5*f* 3212o
19835.64	15	5040.05	*nd* 5_2½_—7*p* 2D212o
19824.93	10	5042.78	*nd* 5_2½_—7*p* 4D312o
19426.10	15	5146.31	6*s*′ ^2^D_1½_—6*p* 2P012o
19370.06	260	5161.20	{7p 4P112o—nd271126p 4S112o—nd5212
19105.35	300	5232.71	6*p* 4P112o—7*s* ^2^P_1½_
19072.11	220	5241.83	{5d 4F412—9p 4D312o6s 4P112—6p 4P112o
19060.64	10	5244.98	6*p* 4P212o—*nd* 5_2½_
18982.85	35	5266.48	*nd* 5.1_1½_—7p 4P112o
18634.52	10	5364.92	6*p* 4S112o*—nd* 5.1_1½_
18348.37	240	5448.59	6*p* 4P212o*—nd* 5.1_1½_
18276.25	110	5470.09	*nd* 5_2½_—7p 4P112o
16213.94	110	6165.85	6*s* ^4^P_0½_—6*p* 4P112o
16192.66	30	6173.96	6*s*′ ^2^D_1½_—6*p* 4D212o
16038.15	400	6233.44	6*p* 4D312o—7*s* ^4^P_2½_
15972.67	115	6258.95	6*p* 2D212o—7*s* ^4^P_2½_
15583.89	250	6415.13	6*p* 4P112o—*nd* 7_1½_
15528.30	280	6438.10	{nd4112—7p 4S112o6p 4P012o—7s 2P1126p 4D112o—7s 4P012
15074.52	125	6631.90	*nd* 11_1½_—6*f* 4212o
15052.50	25	6641.60	*nd* 2_1½_—7*p* 2D212o
15032.73	310	6650.34	6*p* 2D212o—7*s* ^2^P_1½_
14460.38	275	6913.56	{7s 4P212—8p 4S112o6p 2P012o—7s 4P0126p 4D112o—7s 4P112
14287.74	400	6997.10	6*p* 2P212o—7*s* ^4^P_2½_
14272.18	220	7004.73	*nd* 5.1_1½_—4*f* 5212o
14176.65	50	7051.93	*nd* 5.1_1½_—4*f* 6112o
13970.89	35	7156.15	*nd* 5_2½_—4*f* 1212o
13958.54	275	7162.12	{nd5212—4f2312ond5212—4f2.1312o
13869.12	75	7208.30	*nd* 5_2½_—4*f* 5212o
13774.59	65	7257.76	6*p* 4P012o—*nd* 5_1½_
13685.85	200	7304.82	6*p* 4S112o—7*s* ^2^P_1½_
a13387.8	135	7467.4	
13148.85	300	7603.15	5*p*^5^ 2P112o—5*p*^5^ 2P012o
13119.27	} 110	$\{\;\begin{array}{c} 7620.29\\ 7624.26 \end{array}$	6*p* 4P012o—*nd* 7_1½_
13116.03			7*p* 4D312o*—*10*d* ^4^F_4½_
12846.14	} 125	$\{\;\begin{array}{c} 7782.32\\ 7783.74 \end{array}$	6*p* 4P012o*—nd* 8_1½_
12843.83			6*p* 2P012o*—*7*s* ^2^P_0½_
12265.16	50	8150.95	6*p* 4D212o*—*7*d* ^4^P_2½_
12135.81	90	8237.82	*nd* 5.1_1½_—*np* 2112o

a Observed wavelength.

**Table 3:** Wavelengths of Ii in the ultraviolet

Wavelength λvac	Intensity	Wave number	Designation

1876.415	2000	53293.12	2P012o—6*s* ^4^P_0½_
1844.451	15000	54216.66	2P012o—6*s* ^4^P_1½_
1830.380	75000	54633.46	2P112o*—*6*s* ^4^P_2½_
1799.091	5000	55583.61	2P012o—6*s* ^2^P_0½_
1782.758	12000	56092.88	2P112o—6*s* ^2^P_1½_
1702.068	15000	58752.06	2P012o—6*s*′ ^2^D_1½_
1675.174	1500	59695.30	2P012o—*nd* 1_1½_
1642.137	2000	60896.27	2P112o—6*s* ^4^P_0½_
1640.780	2500	60946.62	2P012o—*nd* 2_1½_
1639.106	200	61008.87	2P012o—*nd* 4_1½_
1617.604	5000	61819.81	2P112o—6*s* ^4^P_1½_
1593.580	5000	62751.78	2P012o—*nd* 5.1_1½_
1582.610	1500	63186.76	2P112o—6*s* ^2^P_0½_
1545.794	80	64691.68	2P012o—7*s* ^2^P_1½_
1526.448	2500	65511.57	2P012o—*nd* 6_1½_
1518.047	15000	65874.10	2P012o*—nd* 7_1½_
1514.678	5000	66020.64	2P112o—6*s*′ ^2^D_2½_
1514.323	2000	66036.13	2P012o—*nd* 8_1½_
1507.041	5000	66355.21	2P112o—6*s*′ ^2^D_1½_
1492.888	5000	66984.25	2P012o—*nd* 11_1½_
1485.918	1000	67298.45	2P112o—*nd* 1_1½_
1465.828	2500	68220.83	2P012o—*nd* 16_1½_
1459.145	4000	68533.28	2P012o—*nd* 19_1½_
1458.794	2500	68549.77	2P112o—*nd* 2_1½_
1457.981	10000	68588.00	2P112o—*nd* 3_2½_
1457.470	} 5000	$\{\;\begin{array}{c} 68612.02\\ 68615.84 \end{array}$	2P112o*—nd* 4_1½_
1457.389	5000		2P112o—*nd* 4.1_1½_
1453.179	5000	68814.63	2P012o—*nd* 19.1_0½_
1446.260	5000	69143.83	2P012o*—nd* 20_0½_
1429.539	800	69952.62	2P012o*—*8*s* ^2^P_1½_
1425.490	8000	70151.32	2P112o—*nd* 5_2½_
1421.364	2000	70354.93	2P112o*—nd* 5.1_1½_
1412.180	200	70812.51	2P012o—7*s* ^4^P_0½_
1402.793	} 15	$\{\;\begin{array}{c} 71286.35\\ 71288.01 \end{array}$	2P012o—*nd* 21.2_0½_
1402.758			2P012o—7*s* ^4^P_1½_
1400.014	2000	71427.84	2P012o*—nd* 22_1½_
1395.049	30	71682.11	2P012o—7*s* ^2^P_0½_
1392.898	2000	71792.77	2P012o—*nd* 24_1½_
1390.750	3000	71903.44	2P112o—7*s* ^4^P_2½_
1383.225	4000	72294.83	2P112o—7*s* ^2^P_1½_
1382.284	1200	72344.05	2P012o—9*s* ^2^P_1½_
1368.217	2500	73087.83	2P012o—*nd* 26_1½_
1367.714	2500	73114.72	2P112o—*nd* 6_1½_
1366.506	800	73179.34	2P012o—*nd* 27_1½_
1361.111	3000	73469.39	2P012o*—nd* 28.2_1½_
1360.965	5000	73477.25	2P112o—*nd* 7_1½_
1357.971	3000	73639.28	2P112o—*nd* 8_1½_
1355.542	2000	73771.21	2P012o*—nd* 29_1½_
1355.099	5000	73795.35	2P112o—*nd* 9_1½_
1350.206	600	74062.75	2P012o*—nd* 30_0½_
1349.691	10	74091.05	2P012o*—nd* 31.1_1½_
1348.903	800	74134.29	2P012o*—nd* 32_1½_
1348.768	40	74141.74	2P012o*—nd* 32.1_0½_
1343.626	1000	74425.46	2P012o*—nd* 32.2_0½_
1342.449	60	74490.73	2P012o—*nd* 33_0½_
1341.264	100	74556.55	2P012o*—nd* 34_0½_
1340.709	1500	74587.40	2P112o*—nd* 11_1½_
1339.903	800	74632.28	2P012o*—nd* 35_0½_
*a* 1338.210	20	74726.7	
*b* 1336.478	1000	74823.52	{ 2P112o—nd13212 2P012o—nd36.1012
1335.238	200	74893.04	2P012o*—nd* 38_0½_
1333.232	5	75005.69	2P012o*—nd* 38.2_1½_
*a* 1330.714	30	75147.6	
1330.189	2000	75177.26	2P112o*—nd* 14_2½_
1325.463	10	75445.32	2P012o*—*6*s*″ ^2^S_0½_
1318.844	30	75823.98	2P112o*—nd* 16_1½_
1317.542	3000	75898.91	2P112o*—nd* 17_2½_
1313.947	3000	76106.57	2P112o*—nd* 18_2½_
1313.432	1500	76136.43	2P112o*—nd* 19_1½_
1302.983	3000	76746.98	2P112o*—nd* 20_0½_
1300.335	10000	76903.28	2P112o*—nd* 21_2½_
*a* 1299.012	50	76981.6	
1291.143	300	77450.76	2P112o—8*s* ^4^P_2½_
1289.395	3000	77555.77	2P112o—8*s* ^2^P_1½_
1275.255	1500	78415.66	2P112o—7*s* ^4^P_0½_
1267.596	} 600	$\{\;\begin{array}{c} 78889.50\\ 78891.16 \end{array}$	2P112o*—nd* 21.2_0½_
1267.569			2P112o—7*s* ^4^P_1½_
1266.731	150	78943.37	2P112o—6*d* ^4^P_2½_
1265.326	40	79030.99	2P112o*—nd* 22_1½_
1261.269	800	79285.26	2P112o—7*s* ^2^P_0½_
1259.510	3000	79395.92	2P112o*—nd* 24_1½_
1259.153	2500	79418.49	2P112o*—nd* 25_2½_
1251.335	600	79914.68	2P112o—9*s* ^4^P_2½_
1250.826	400	79947.20	2P112o—9*s* ^2^P_1½_
1249.969	15	80001.95	2P112o*—nd* 25.1_0½_
1239.463	70	80680.12	2P112o—7*d* ^4^P_2½_
1239.296	70	80690.98	2P112o*—nd* 26_1½_
1239.249	70	80694.00	2P112o*—nd* 26.1_0½_
1237.892	300	80782.49	2P112o*—nd* 27_1½_
1237.231	200	80825.65	2P112o*—nd* 27.1_2½_
1236.362	400	80882.46	2P112o*—nd* 28.1_2½_
*a* 1233.517	50	81069.0	
1233.463	300	81072.54	2P112o*—nd* 28.2_1½_
*a* 1232.914	10	81108.7	
1230.732	400	81252.48	2P112o—10*s* ^4^P_2½_
1228.888	500	81374.36	2P112o*—nd* 29_1½_
*a* 1228.041	100	81430.5	
1224.856	400	81642.25	2P112o*—nd* 31_1½_
1224.501	300	81665.90	2P112o*—nd* 30_0½_
1224.077	} 600	$\{\;\begin{array}{c} 81694.20\\ 81696.06 \end{array}$	2P112o*—nd* 31.1_1½_
1224.049			2P112o—8*d* ^4^P_2½_
1223.430	100	81737.44	2P112o*—nd* 32_1½_
*a* 1219.327	10	82012.4	
1219.087	70	82028.61	2P112o*—nd* 32.2_0½_
*a* 1218.909	70	82040.6	
*a* 1218.711	100	82053.9	
1218.411	200	82074.10	2P112o—11*s* ^4^P_2½_
1218.118	40	82093.88	2P112o*—nd* 33_0½_
1217.142	150	82159.70	2P112o*—nd* 34_0½_
*c* 1216.021	?	82235.43	2P112o*—nd* 35_0½_
*a* 1214.631	50	82329.5	
1213.627	40	82397.62	2P112o*—nd* 36_2½_
1213.199	60	82426.68	2P112o*—nd* 36.1_0½_
1212.242	60	82491.80	2P112o*—nd* 37_2½_
1212.177	60	82496.19	2P112o*—nd* 38_0½_
1210.880	10	82584.58	2P112o—*nd.* 38.1_2½_
1210.524	60*Z*	82608.84	2P112o*—nd* 38.2_1½_
1210.050	60	82641.21	2P112o*—nd* 39_2½_
*a* 1208.466	50	82749.5	
*a* 1207.964	20	82783.9	
1206.988	10	82850.87	2P112o*—nd* 40_2½_
*a* 1205.430	25	82957.9	
1204.116	40	83048.47	2P112o—6*s*″ ^2^S_0½_
*a* 1201.348	30	83239.8	
*a* 1200.946	30	83267.7	
*a* 1200.711	25	83284.0	
*a* 1196.786	10	83557.1	
*a* 1195.288	15	83661.8	

a Wavelengths measured by C. H. Corliss and W. C. Martin.

b Also I ii.

c Masked by I ii and Ly*α*.

**Table 4:** Zeeman effect of I i

Wavelength	Magnetic patterns

11236.56	(0.117, **0.356)** 1.240, **1.479,** 1.721
11020.60	**(0.123,** 0.370, 0.643) **0.629,** 0.844, 1.044
11017.14	(0.524) …
10685.82	(0.754) …
10466.54	**(0.079,** 0.240) **0.976,** 1.147, 1.299, 1.488
10435.34	(0.257) …
10416.61	(0.428. **1.230)** 0.369, **1.187,** 1.985
10391.74	(0.249) 1.042, **1.563**
10375.20	(0.000*w*) 0.943*w A*
10375.20	**(0.059,** 0.252, 0.419, 0.603, 0.775) **1.034,** 1.211, 1.383, 1.586, 1.753, 1.897
10325.90	(0.194) 1.163, 1.441, **1.805**
10238.82	(0.086) **1.298,** 1.494
10172.91	(0.000*w*) 0.907
10158.64	(0.213) 1.348 *w*
10141.83	(0.370) 0.482, **1.152**
10131.16	(0.275) 1.095, **1.612**
10003.05	(0.118) 1.500, **1.747**
9963.30	(0.000*w*) 1.053*w A*
9842.75	(0.141, **0.449)** 1.184, **1.472,** 1.785
9813.53	**(0.179,** 0.553) **0.702,** 1.077, 1.445
9800.89	(0.170) 0.952
9749.20	(0.000) 1.178
9744.83	(0.141) …
9731.73	(0.150) 1.502
9725.47	(0.245) **0.798,** 1.290
9653.06	(0.000) 1.525
9649.61	(0.000) 0.982 *A*
9598.22	(0.000) 1.053
9466.34	(0.282) 1.818
9427.15	**(0.096,** 0.274, 0.451) **0.879,** 1.050, 1.223, 1.405
9426.71	(0.654) 1.886
9335.05	**(0.118,** 0.348) **1.005,** 1.250, 1.500, 1,738
9321.95	(0.395) 1.216, **1.991**
9227.74	(0.114. **0.300)** 1.294, **1.505,** 1.705
9180.20	(0.157) 1.114
9156.91	(0.055. **0.585)** 1.531, **1.068**
9128.03	**(0.514,** 1.401) **0.647,** 1.613
9113.91	(0.000) 1.385
9098.86	(0.170, **0.504)** 1.118, **1.460,** 1.790
9087.16	(0.476) 0.650, **1.009,** 1.317
9079.34	(0.174, 0.478, **0.868)** 0.682, 1.036, **1.372,** 1,738, 2.117
9058.33	**(0.090,** 0.268, 0.455) **0.934,** 1.107, 1.294, 1.503, 1.698
9022.40	(0.622) **0.717,** 1.939
8993.13	**(0.196,** 0.552) **0.638,** 1.033, 1.419
8969.04	(0.289, **0.853)** 1.311
8964.69	(0.000) 1.098
8925.97	(0.444, **0.743)** 0.821, 1.102, **1.372,** 1.676, 1.971
8898.50	(0.157, **0.452)** 1.185, **1.492,** 1.793
8857.50	}P–B
8853.80	
8853.24	
8847.14	(0.427) 0.840
8816.65	(0.362) 1.083, **1.389,** 1.458
8748.22	(0.266) 1.338
8700.80	(0.269) 0.910, 1.091
8664.95	(0.062) 1.198
8636.40	(0.098) 1.477
8545.52	(0.098) 1.268 *A*
8486.11	**(0.072,** 0.224, 0.375) **1.038**
8393.30	(0.640) **0.659,** 1.951
8391.70	**(0.067,** 0.184) **1.049,** 1.143, 1.303
8260.04	(0.218*w*) 1.284*w*
8251.08	(0.389) 1.183
8240.05	(0.0 *W*) 1.043 *A*
8222.57	(0.622) **0.721,** 1.950
8169.38	**(0.117,** 0.325) 0.762
8105.60	(0.000) 1.396
8090.76	**(0.111,** 0.382) **0.809,** 1.029, 1.264
8065.70	**(0.000) 1.343**
8043.74	**(0.082,** 0.242) 1.855 *B*
8039.85	(0.339) …
8023.01	(0.000) 1.524
8003.63	(0.714) 1.868
7974.48	(0.128), 0.616, **0.920**
7969.48	(0.106) 1.536
7955.90	(0.234) …
7944.85	(0.000) 0.929
7700.20	**(0.074,** 0.265) 0.959
7671.01	(0.330) 1.223
7604.88	**(0.068,** 0.212) 1.376
7588.60	(0.180) 1.053
7556.65	(0.037) 1.598
7554.18	(0.182) 1.313, **1.418**
7531.81	(0.000) 1.480
7490.52	(0.000) 1.244
7468.99	**(0.045,** 0.143, 0.237) **1.006,** 1.088, 1.187
7446.37	(0.344) …
7416.48	(0.523) 0.606, **0.989,** 1.346
7413.60	(0.000*w*) 0.933*w*
7411.20	(0.000) 1.186
7410.50	(0.000*w*) 1.120
7402.06	(0.000*W*) 0.993 *A*
7384.08	(0.000) 1.379
7305.43	(0.430) 1.167
7259.98	(0.000) 1.393
7258.06	(0.072) 1.196
7237.84	**(0.128,** 0.361, 0.599) 0.590
7236.78	**(0.060,** 0.198, 0.341, 0.454) **0.807**,0.938, 1.050
7231.82	(0.362) 1.159
7227.30	(0.606) …
7192.52	(0.390) 0.932, **1.758**
7191.66	(0.000) 1.589
7164.79	**(0.130,** 0.418) **0.967,** 1.251
7148.63	(…) 1.402
7142.06	**(0.075,** 0.246, 0.417) **0.947,** 1.106, 1.277
7122.05	(0.247, **0.386)** … **1.293,** 1.435
7120.05	(0.206, **0.616)** 0.965, **1.437,** 1.838
7085.05	(0.000) 1.171
7063.59	(0.501) 1.092
6789.23	(0.553) 0.920
6732.03	(0.000 *W)* 0.939 *w*
6698.46	(0.000) 1.492 *A*
6697.29	**(0.112,** 0.368, 0.643) ….
6662.10	(0.243 *w*) 1.325 *w*
6661.11	(0.092) 1.530
6619.66	(0.000 *W)* 1.553 *A*
6585.27	(0.275) 0.670. **0.898**
6583.75	(0.000 *W*) 1.028 *A*
6570.38	(0.165) 1.086
6566.49	**(0.086,** 0.275) **0.824,** 0.983, 1.135
6564.80	(0.285) …
6560.82	(0.143 *w*) 1.204
6488.10	(0.078) 1.310, **1.450**
6455.00	}P–B
6434.49	
6433.28	
6415.70	(0.000 *w*, 0.303) 1.154 *A*
6411.22	(0.339) 1.140
6371.68	**(0.142,** 0.354) **1.007,** 1.246, 1.500
6367.28	(0.217, **0.707)** 0.604, **1.090,** 1.585
6359.16	(0.000) 1.375
6339.44	(0.000 *w*, 0.186, 0.377) **0.930,** 1.113
6337.85	(0.217, **0.367)** 1.598
6330.37	(0.228) 1.575
6313.13	(0.195, **0.371)** 1.257
6297.00	(0.000 *w*) 1.540
6293.98	(0.000) 1.350
6244.48	(0.000 *W*) …
6233.50	(0.278) …
6213.10	(0.000 *W*) 1.097 *A*
6191.88	(0.000 *W*) 1.050
6115.97	(0.000 *w*, 0.205) 1.529 *B*
6082.43	(0.000) 1.388
6073.46	}P–B
6055.96	
6055.03	
6053.49	(0.000) 0.888
6024.08	(0.172, **0.406)** 1.213, **1.489,** 1.759
5981.26	(0.124) 1.345
5960.40	**(0.358,** 1.036) **0.000***d*, 0.643, 1.273
5894.03	(0.000) 1.346
5882.24	(0.000*w*) 1.699
5780.65	(0.122) 1.261, **1.505**
5764.33	**(0.131,** 0.349) 1.243, 1.473, 1.749, **2.014**
5743.90	(0.478) …
5586.36	(0.365) 1.280, **1.483,** 1.707
5500.95	**(0.121,** 0.416) 1.443, 1.795, **2.070**
5297.17	(0.000) 1.595
5273.72	(0.289) 1.272, **1.447,** 1.657
5265.69	(0.000) 0.901
5234.57	(0.000) 1.038 *A*
5204.15	(0.074) **1.298,** 1.434
5145.52	(0.000*w*) 0.885 *A*
5119.29	(0.116) 1.415
4916.94	(0.217) 1.538
4902.00	(0.000*w*) 1.129 *A*
4862.96	(0.216, 0.549, **0.913)** 0.712, 1.078, **1.421,** 1.749, 2.096
4862.32	(0.000) 1.073
4850.51	}P–B
4850.35	
4827.57	(0.495) 0.990
4763.31	(0.000) 1.630
4700.88	(0.000*W*) 0.958*A*
4690.49	(0.518) 0.841, **1.433, …**
4478.56	(0.000) 1.377
4409.12	(…) 0.626
4408.01	**(0.238,** 0.699, 1.228) **0.000,** 0.328, 0.877, 1.361, 1.914
4406.54	(…) 1.368
4399.01	(0.000) 0.956
4392.09	(0.000*w*) 0.715*A*
4321.84	(0.000) 1.368
4234.54	(0.000*d*) 1.243, **1.595**
4209.82	(…) 1.198
4203.72	(0.000) 1.786
4148.41	(0.000) 1.515
4134.15	(0.000) 1.442
4129.21	(0.000) 1.122
2061.63	(0.335) 1.005, **1.669**

**Table 5:** Predicted terms of I i

Electron configuration	Terms		

5*s*^2^ 5*p*^5^	^2^P°		

5*s* 5*p*^6^	^2^S		

5*s*^2^ 5*p*^4^ (I ii)	^3^P	^1^D	^1^S

5*s*^2^ 5*p*^4^ *ns*	^2^P ^4^P	^2^D	^2^S
5*s*^2^ 5*p*^4^ *np*	^2^S°, ^2^P°, ^2^D°, ^4^S°, ^4^P°, ^4^D°	^2^P°, ^2^D°, ^2^F°	^2^P°
5*s*^2^ 5*p*^4^ *nd*	^2^P, ^2^D, ^2^F, ^4^P, ^4^D, ^4^F	^2^S, ^2^P, ^2^D, ^2^F, ^2^G	^2^D
5*s*^2^ 5*p*^4^ *nf*	^2^D°, ^2^F°, ^2^G°, ^4^D°, ^4^F°, ^4^G°	^2^P°, ^2^D°, ^2^F°, ^2^G°, ^2^H°	^2^F°

**Table 6:** Odd terms of I i

Electron configuration	Term symbol	Level	Δ*v*	Observed *g*

5*s*^2^ 5*p*^5^	$\left\{ \begin{array}{r} 5p^{5}{\;}^{2}P_{1½}^{o}\\ {\;}^{2}P_{0½}^{o} \end{array}\right.$	0.00	−7603.15	
		7603.15		0.673
5*s*^2^ 5*p*^4^(^3^P)6*p*	$\left\{ \begin{array}{r} 6p{\;}^{4}P_{2½}^{o}\\ {\;}^{4}P_{1½}^{o}\\ {\;}^{4}P_{0½}^{o} \end{array}\right.$	64906.34	−2155.78 1205.16	1.524
		67062.12		1.415
		65856.96		1.556
5*s*^2^ 5*p*^4^(^3^P)6*p*	$6p{\;}^{4}S_{1½}^{o}$	64990.01		1.619
5*s*^2^ 5*p*^4^(^3^P)6*p*	$\{\;\begin{array}{c} 6p{\;}^{2}D_{2½}^{o}\\ {\;}^{2}D_{1½}^{o} \end{array}$	65644.49	−7162.72	1.217
		72807.21		1.316
5*s*^2^ 5*p*^4^(^3^P)6*p*	$\left\{ \begin{array}{r} 6p{\;}^{4}D_{3½}^{o}\\ {\;}^{4}D_{2½}^{o}\\ {\;}^{4}D_{1½}^{o}\\ {\;}^{4}D_{0½}^{o} \end{array}\right.$	65670.00	−6859.17 552.43 −1410.44	1.420
		72529.17		1.370
		71976.74		1.317
		73387.18		1.137
5*s*^2^ 5*p*^4^(^3^P)6*p*	$\{\;\begin{array}{c} 6p{\;}^{2}P_{0½}^{o}\\ {\;}^{2}P_{1½}^{o} \end{array}$	71501.52	1553.04	1.239
		73054.56		1.329
5*s*^2^ 5*p*^4^(^3^P)6*p*	$6p{\;}^{2}S_{0½}^{o}$	71813.97		1.377
5*s*^2^ 5*p*^4^( )*np*	$np\;1_{1½}^{o}$	72875.75		
5*s*^2^ 5*p*^4^(^8^P)7*p*	$\left\{ \begin{array}{r} 7p{\;}^{4}P_{2½}^{o}\\ {\;}^{4}P_{1½}^{o}\\ {\;}^{4}P_{0½}^{o} \end{array}\right.$	74965.77	−655.64 318.28	1.472
		75621.41		1.483
		75303.13		1.53
5*s*^2^ 5*p*^4^(^3^P)7*p*	$7p{\;}^{4}S_{1½}^{o}$	75049.57		1.506
5*s*^2^ 5*p*^4^(^3^P)7*p*	$\left\{ \begin{array}{r} 7p{\;}^{4}D_{2½}^{o}\\ {\;}^{2}D_{1½}^{o} \end{array}\right.$	75191.37	−7232.81	1.24
		82424.18		1.27
5*s*^2^ 5*p*^4^(^3^P)7*p*	$\left\{ \begin{array}{r} 7p{\;}^{4}D_{3½}^{o}\\ {\;}^{4}D_{2½}^{o}\\ {\;}^{4}D_{1½}^{o}\\ {\;}^{4}D_{0½}^{o} \end{array}\right.$	75194.10	−6839.70 310.82	1.42
		82033.80		
		81722.98		1.39
		…		
5*s*^2^ 5*p*^4^(^3^P)7*p*	$\left\{ \begin{array}{r} 7p{\;}^{2}P_{0½}^{o}\\ {\;}^{2}P_{1½}^{o} \end{array}\right.$	78780.04	−3651.16	1.48
		82431.20		
5*s*^2^ 5*p*^4^(^3^P)7*p*	$7p{\;}^{2}S_{0½}^{o}$	81506.80		
5*s*^2^ 5*p*^4^(^3^P)4*J*	$\left\{ \begin{array}{r} 4f\;0_{1½}^{o}\\ 4f\;0.1_{0½}^{o}\\ 4f\;1_{2½}^{o}\\ 4f\;2_{3½}^{o}\\ 4f\;2.1_{3½}^{o}\\ 4f\;3_{3½}^{o}\\ 4f\;4_{1½}^{o}\\ 4f\;5_{2½}^{o}\\ 4f\;5.1_{1½}^{o}\\ 4f\;6_{1½}^{o} \end{array}\right.$	77297.15		
		77303.58		
		77307.47		
		77313.12		
		77313.76		
		77320.66		
		77356.76		
		77359.62		
		77404.49		
		77406.86		
5*s*^2^ 5*p*^4^(^1^D) 6*p*	$6p^{\prime }{\;}^{2}P_{1½}^{o}$	78415.36		
5*s*^2^ 5*p*^4^( )*np*	$np\;1.1_{2½}^{o}$	78535.48		
5*s*^2^ 5*p*^4^( )*np*	$np\;2_{1½}^{o}$	78592.75		1.00
5*s*^2^ 5*p*^4^(^3^P)8*p*	$\left\{ \begin{array}{r} 8p{\;}^{4}P_{2½}^{o}\\ {\;}^{4}P_{1½}^{o}\\ {\;}^{4}P_{0½}^{o} \end{array}\right.$	78732.31	−492.47 21.67	1.454
		79224.78		1.30
		79203.11		
5*s*^2^ 5*p*^4^(^3^P)8*p*	$8p{\;}^{4}S_{1½}^{o}$	78815.61		1.71
5*s*^2^ 5*p*^4^(^3^P)8*p*	$8p{\;}^{2}D_{2½}^{o}$	78854.86		1.11
5*s*^2^ 5*p*^4^(^3^P)8*p*	$8p{\;}^{4}D_{3½}^{o}$	79003.70		1.37
5*s*^2^ 5*p*^4^(^3^P)8*p*	$8p{\;}^{2}P_{0½}^{o}$	79701.73		1.02
5*s*^2^ 5*p*^4^(^3^P)5*f*	$\left\{ \begin{array}{r} 5f\;1_{1½}^{o}\\ 5f\;2_{0½}^{o}\\ 5f\;3_{2½}^{o}\\ 5f\;4_{3½}^{o}\\ 5f\;4.1_{3½}^{o}\\ 5f\;4.2_{3½}^{o}\\ 5f\;5_{1½}^{o} \end{array}\right.$	79835.03		
		79840.23		
		79844.58		
		79847.88		
		79853.40		
		79865.20		
		79881.74		
5*s*^2^ 5*p*^4^( )*np*	$\left\{ \begin{array}{r} np\;3_{1½}^{o}\\ np\;4_{2½}^{o} \end{array}\right.$	80039.94		
		80125.57		
5*s*^2^ 5*p*^4^(^3^P)9*p*	$\left\{ \begin{array}{r} 9p{\;}^{4}P_{2½}^{o}\\ 9p{\;}^{4}S_{1½}^{o}\\ 9p{\;}^{2}D_{2½}^{o}\\ 9p{\;}^{4}D_{3½}^{o} \end{array}\right.$	80624.45		
		80720.85		
		80797.95		
		80945.44		1.32
5*s*^2^ 5*p*^4^(^3^P)6*f*	$\left\{ \begin{array}{r} 6f\;1_{2½}^{o}\\ 6f\;2_{3½}^{o}\\ 6f\;3_{1½}^{o}\\ 6f\;4_{2½}^{o}\\ 6f\;5_{0½}^{o}\\ 6f\;6_{1½}^{o} \end{array}\right.$	81205.39		
		81207.32		
		81216.75		
		81219.30		
		81226.02		
		81227.38		
5*s*^2^ 5*p*^4^( )*np*	$np\;5_{3½}^{o}$	81693.55		
5*s*^2^ 5*p*^4^(^3^P)10*p*	$\left\{ \begin{array}{r} 10p{\;}^{4}P_{2½}^{o}\\ 10p{\;}^{4}D_{3½}^{o} \end{array}\right.$	81697.54		
		82035.00		
5*s*^2^ 5*p*^4^(^3^P)7*f*	$\left\{ \begin{array}{r} 7f\;1_{2½}^{o}\\ 7f\;2_{3½}^{o} \end{array}\right.$	82026.20		
		82030.35		
5*s*^2^ 5*p*^4^()*np*	$\left\{ \begin{array}{r} np\;6_{2½}^{o}\\ np\;7_{1½}^{o} \end{array}\right.$	82214.04		
		82615.84		

**Table 7:** Even terms of I i

Electron configuration	Term symbol	Level	Δ*v*	Observed *g*

5*s*^2^ 5*p*^4^(^3^P)6*s*	$\left\{ \begin{array}{r} 6S{\;}^{4}P_{2½}\\ {\;}^{4}P_{2½}\\ {\;}^{4}P_{2½} \end{array}\right.$	54633.46	−7186.35 + 923.54	1.576
		61819.81		1.618
		60896.27		2.561
5*s*^2^ 5*p*^4^(^3^P)6*s*	$\left\{ \begin{array}{r} 6s{\;}^{2}P_{1½}\\ {\;}^{2}P_{0½} \end{array}\right.$	56092.88	−7093.88	1.385
		63186.76		0.799
5*s*^2^ 5*p*^4^(^1^D)6*s*	$\left\{ \begin{array}{r} 6s^{\prime }{\;}^{2}D_{2½}\\ {\;}^{2}D_{1½} \end{array}\right.$	66020.64	−334.57	1.258
		66355.21		0.828
5*s*^2^ 5*p*^4^(^1^S)6*s*	6*s*″ ^2^S_0½_	83048.47		
5*s*^2^ 5*p*^4^(^3^P)7*s*	$\left\{ \begin{array}{r} 7s{\;}^{4}P_{2½}\;\\ {\;}^{4}P_{1½}\\ {\;}^{4}P_{0½} \end{array}\right.$	71903.44	−6987.72 + 475.50	
		78891.16		
		78415.66		
5*s*^2^ 5*p*^4^(^3^P)7*s*	$\left\{ \begin{array}{r} 7s{\;}^{2}P_{1½}\\ {\;}^{2}P_{0½} \end{array}\right.$	72294.83	−6990.43	
		79285.26		
5*s*^2^ 5*p*^4^(^3^P)8*s*	$\left\{ \begin{array}{r} 8s{\;}^{4}P_{2½}\\ {\;}^{2}P_{1½} \end{array}\right.$	77450.76		
		77555.77		
*5s*^2^ 5*p*^4^(^3^P)9*s*	$\left\{ \begin{array}{r} 9s{\;}^{4}P_{2½}\\ {\;}^{2}P_{1½} \end{array}\right.$	79914.68		
		79947.20		
5*s*^2^ 5*p*^4^(^3^P) 10*s*	$\left\{ \begin{array}{r} 10s{\;}^{4}P_{2½}\\ {\;}^{2}P_{1½} \end{array}\right.$	81252.48		1.53
		…		
5*s*^2^ 5*p*^4^(^3^P)11*s*	$\left\{ \begin{array}{r} 11s{\;}^{4}P_{2½}\\ {\;}^{2}P_{1½} \end{array}\right.$	82074.10		
		…		
5*s*^2^ 5*p*^4^(^3^P)5*d*	$\left\{ \begin{array}{r} 5d{\;}^{4}D_{3½}\\ {\;}^{4}P_{2½}\\ {\;}^{4}F_{4½}\\ {\;}^{2}F_{3½} \end{array}\right.$	75511.13		1.15
		…		
		75704.09		
		76004.78		
5*s*^2^ 5*p*^4^(^3^P)6*d*	$\left\{ \begin{array}{r} 6d{\;}^{4}D_{3½}\;\\ {\;}^{4}P_{2½}\\ {\;}^{4}F_{4½} \end{array}\right.$	78904.05		
		78943.37		
		79055.01		
5*s*^2^ 5*p*^4^(^3^P)7*d*	$\left\{ \begin{array}{r} 7d{\;}^{4}D_{3½}\;\\ {\;}^{4}P_{2½}\\ {\;}^{4}F_{4½} \end{array}\right.$	80676.17		
		80680.12		
		80772.35		
5*s*^2^ 5*p*^4^(^3^P)8*d*	$\left\{ \begin{array}{r} 8d{\;}^{4}D_{3½}\;\\ {\;}^{4}P_{2½}\\ {\;}^{4}F_{4½} \end{array}\right.$	81689.02		
		81696.06		
		81760.58		
5*s*^2^ 5*p*^4^(^3^P)9*d*	$\left\{ \begin{array}{r} 9d{\;}^{4}D_{3½}\;\\ {\;}^{4}P_{2½}\\ {\;}^{4}F_{4½} \end{array}\right.$	82289.46		
		…		
		82374.21		
5*s*^2^ 5*p*^4^(^3^P)10*d*	$\left\{ \begin{array}{r} 10d{\;}^{4}D_{3½}\;\\ {\;}^{4}P_{2½}\\ {\;}^{4}F_{4½} \end{array}\right.$	…		
		…		
		82818.36		
5*s*^2^ 5*p*^4^( )*nd*	$\{\;\begin{array}{l} nd\;1_{1½}\\ nd\;2_{1½}\\ nd\;3_{2½}\\ nd\;4_{1½}\\ nd\;4.1_{1½}\\ nd\;5_{2½} \end{array}$	67298.45		
		68549.77		
		68588.00		
		68612.02		
		68615.84		
		70151.32		
5*s*^2^ 5*p*^4^( )*nd*	$\{\;\begin{array}{l} nd\;5.1_{1½}\\ nd\;6_{1½}\\ nd\;7_{1½}\\ nd\;8_{1½}\\ nd\;9_{2½}\\ nd\;10_{3½} \end{array}$	70354.93	1.21	
		73114.72		
		73477.25		
		73639.28		
		73795.35		
		73977.68		
5*s*^2^ 5*p*^4^( )*nd*	$\{\;\begin{array}{l} nd\;11_{1½}\\ nd\;13_{2½}\\ nd\;14_{2½}\\ nd\;15_{0½}\\ nd\;16_{1½} \end{array}$	74587.40	0.78	
		74823.48		
		75177.26	1.26	
		75714.44	0.80	
		75823.98	1.41	
5*s*^2^ 5*p*^4^( )*nd*	$\{\;\begin{array}{l} nd\;17_{2½}\\ nd\;18_{2½}\\ nd\;19_{1½}\\ nd\;19.1_{0½}\\ nd\;20_{0½} \end{array}$	75898.91	1.14	
		76106.57	1.23	
		76136.43	1.04	
		76417.78		
		76746.98		
5*s*^2^ 5*p*^4^( )*nd*	$\{\;\begin{array}{l} nd\;21_{2½}\\ nd\;21.1_{3½}\\ nd\;21.2_{0½}\\ nd\;22_{1½}\\ nd\;23_{3½} \end{array}$	76903.28		
		76935.98		
		78889.50		
		79030.99	1.23	
		79150.55		
	$\{\;\begin{array}{l} nd\;24_{1½}\\ nd\;25_{2½}\\ nd\;25.1_{0½}\\ nd\;26_{1½}\\ nd\;26.1_{0½} \end{array}$	79395.92	1.42	
		79418.49	1.17	
		80001.95		
		80690.98		
		80694.00		
	$\{\;\begin{array}{l} nd\;27_{1½}\\ nd\;27.1_{2½}\\ nd\;28_{3½}\\ nd\;28.1_{2½}\\ nd\;28.2_{1½} \end{array}$	80782.49	1.46	
		80825.65		
		80869.05		
		80882.46		
		81072.54		
	$\{\;\begin{array}{l} nd\;29_{1½}\\ nd\;30_{0½}\\ nd\;31_{1½}\\ nd\;31.1_{1½}\\ nd\;32_{1½} \end{array}$	81374.36		
		81665.90		
		81642.25		
		81694.20		
		81737.44		
	$\{\;\begin{array}{l} nd\;32.1_{0½}\\ nd\;32.2_{0½}\\ nd\;33_{0½}\\ nd\;34_{0½}\\ nd\;35_{0½} \end{array}$	81744.89		
		82028.61		
		82093.88		
		82159.70		
		82235.43		
	$\{\;\begin{array}{l} nd\;36_{2½}\\ nd\;36.1_{0½}\\ nd\;37_{2½}\\ nd\;38_{0½} \end{array}$	82397.62		
		82426.68		
		82491.80		
		82496.19		
	$\{\;\begin{array}{l} nd\;38.1_{2½}\\ nd\;38.2_{1½}\\ nd\;39_{2½}\\ nd\;40_{2½} \end{array}$	82584.58		
		82608.84		
		82641.21		
		82850.87		

**Table 8:** Series of I i

*n*	*ns* ^4^P_2½_		*np* 4P412o		*nd* ^4^F_4½_		*nf* X_3½_	

	Current term	Rydberg denominator	Current term	Rydberg denominator	Current term	Rydberg denominator	Current term	Rydberg denominator

4							7019	3.95402
5					8636	3.56468	4492	4.94261
6	29707	1.92197	19434	2.37627	5285	4.55674	3133	5.91829
7	12437	2.97043	9374	3.42148	3568	5.54580	2310	6.89240
8	6889	3.99116	5608	4.42357	2579	6.52305		
9	4425	4.97989	3716	5.43424	1955	7.49209		
10	3088	5.96126	2643	6.44359				
11	2266	6.95900						
	*α*= −4.00389		*α*= −3.51112		*α*= −1.48320		*α*= −0.11660	
	*β*= − 2.52065		*β*= − 3.98279		*β*= + 1.15995		*β*= + 1.16647	

**Table 9:** Continua in the spectrum of iodine

Wavelengths of maxima	Wave numbers of maxima	Intensity	Δ*v*	Remarks

4625	21616	1000	1715	Band extends from 4820 to 4400 A
				Minimum at 4360 A
4285	23331	100		Band extends from 4325 to 4240 A
3525	28361	25	325	Minimum at 3510 A
3485	28686	50	503	Minimum at 3475 A
3425	29189	500	1290	Band extends from 3470 to 3380 A
				Minimum at 3340 A
3280	30479	5	206	Minimum at 3270 A
3258	30685	10	228	Minimum at 3245 A
3234	30913	25	211	Minimum at 3221 A
3212	31124	50	205	Minimum at 3199 A
3191	31329	25	208	Minimum at 3177 A
3170	31537	10	281	Minimum at 3155 A
3142	31818	5	193	Minimum at 3136 A
3123	32011	20	217	Minimum at 3114 A
3102	32228	15	230	Minimum at 3091 A
3080	32458	10	211	Minimum at 3074 A
3062	32649	20	215	Minimum at 3053 A
3042	32864	25	184	Minimum at 3032 A
3025	33048	15	187	Minimum at 3016 A
3008	33235	20	234	Minimum at 2997 A
2987	33469	20	214	Minimum at 2976 A
2968	33683	10	182	Minimum at 2959 A
2952	33861	15	173	Minimum at 2943 A
2937	34038	5		

## Footnotes

1 Figures in brackets indicate the literature references at the end of this paper.
